# The origin and fate of volatile elements on Earth revisited in light of noble gas data obtained from comet 67P/Churyumov-Gerasimenko

**DOI:** 10.1038/s41598-020-62650-3

**Published:** 2020-04-02

**Authors:** David V. Bekaert, Michael W. Broadley, Bernard Marty

**Affiliations:** Centre de Recherches Pétrographiques et Géochimiques, UMR 7358 CNRS - Université de Lorraine, 15 rue Notre Dame des Pauvres, BP 20, 54501 Vandoeuvre-lès-Nancy, France

**Keywords:** Atmospheric chemistry, Geochemistry

## Abstract

The origin of terrestrial volatiles remains one of the most puzzling questions in planetary sciences. The timing and composition of chondritic and cometary deliveries to Earth has remained enigmatic due to the paucity of reliable measurements of cometary material. This work uses recently measured volatile elemental ratios and noble gas isotope data from comet 67P/Churyumov-Gerasimenko (67P/C-G), in combination with chondritic data from the literature, to reconstruct the composition of Earth’s ancient atmosphere. Comets are found to have contributed ~20% of atmospheric heavy noble gases (i.e., Kr and Xe) but limited amounts of other volatile elements (water, halogens and likely organic materials) to Earth. These cometary noble gases were likely mixed with chondritic - and not solar - sources to form the atmosphere. We show that an ancient atmosphere composed of chondritic and cometary volatiles is more enriched in Xe relative to the modern atmosphere, requiring that 8–12 times the present-day inventory of Xe was lost to space. This potentially resolves the long-standing mystery of Earth’s “missing xenon”, with regards to both Xe elemental depletion and isotopic fractionation in the atmosphere. The inferred Kr/H_2_O and Xe/H_2_O of the initial atmosphere suggest that Earth’s surface volatiles might not have been fully delivered by the late accretion of volatile-rich carbonaceous chondrites. Instead, “dry” materials akin to enstatite chondrites potentially constituted a significant source of chondritic volatiles now residing on the Earth’s surface. We outline the working hypotheses, implications and limitations of this model in the last section of this contribution.

## Introduction

Earth’s early atmosphere experienced a complex history of impact erosion, mantle outgassing and late additions during periods of heavy asteroid and cometary bombardments^1^. The purported Moon forming impact is speculated to have removed a significant fraction of the proto-Earth’s atmosphere^2^ and resulted in a deep magma ocean estimated to have lasted for several million years^3^. Although the presence of a Late Heavy Bombardment (LHB), inferred from the lunar cratering record, has been cast into doubt^4^, the net flux of extraterrestrial materials crossing Earth’s orbit was arguably higher during the infancy of the solar system^5^. Given its inner solar system origin, the Earth is expected to have grown dry. Volatile-rich bodies (carbonaceous chondrites - hereafter CC - and/or comets) striking the Earth after core formation have been suggested as the suppliers of volatiles that formed the terrestrial oceans and atmosphere, as well as delivering primitive organic materials^6^. The late accretion of chondritic material to Earth after formation of the Moon and core segregation, commonly referred to as the terrestrial “late veneer” (~0.5wt.% of the Earth), is required to account for the high and unfractionated abundances of highly siderophile elements in the terrestrial mantle^7^. However, the final stages of Earth’s accretion have been argued to be predominantly derived from the inner solar system and to resemble enstatite chondrites (EC), which are considered to be our best isotopic analogues for the main building blocks of the Earth^8,9^. The atmosphere (ATM) might therefore be considered as a complex mix between inherited solar and chondritic volatiles, plus later introduction of chondrites and/or comets^10^. To date, deciphering whether the majority of the Earth’s volatiles were derived from cometary or chondritic sources has been hampered by the lack of reliable measurements of cometary material.

**Figure 1:**
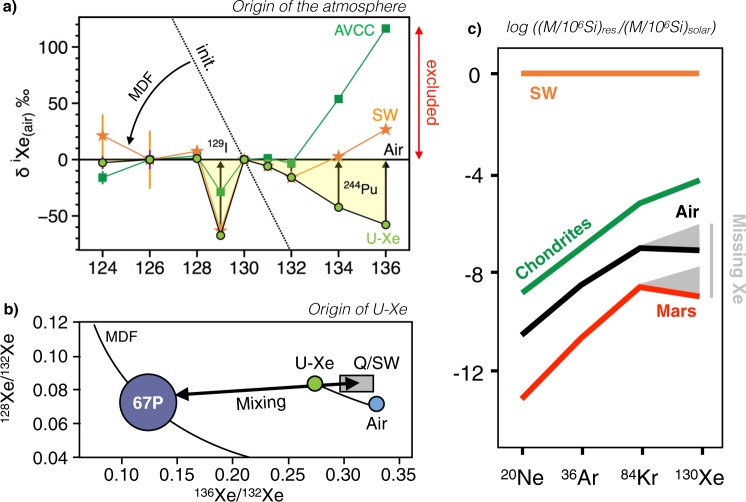
Origin of atmospheric Xe’s precursor (U-Xe). (**a**) AVerage Carbonaceous Chondrite Xe **(**AVCC-Xe)^11^, solar wind xenon (SW^32^) and U-Xe^11^ signatures mass-dependently fractionated to the extent where their ^126^Xe/^130^Xe matches the Air-Xe value. Initial U-Xe composition is shown by dotted line. It appears that post-MDF AVCC-Xe and SW-Xe compositions contribute more ^136^Xe relative to ^130^Xe than the atmosphere actually contains, therefore excluding chondritic and solar components as the only sources of atmospheric volatiles^11^. The mass-dependently fractionated signature of U-Xe exhibits deficits in ^129^Xe and ^131–134^Xe that correspond to latter additions of radiogenic and fissiogenic contributions from ^129^I and ^244^Pu, respectively^11^. (**b**) Mixing diagram between cometary and Q^33^/SW-Xe^32^ components to account for the origin of U-Xe in the terrestrial atmosphere (modified from^12^), as modelled by the thick arrow. Subsequent evolution from U-Xe to Air by MDF is represented by the solid curve. (**c**) Comparison of the abundance patterns of noble gases in the Sun, in volatile-rich primitive chondrites and in the atmospheres of Earth and Mars (modified after^17,18^). Earth and Mars are depleted in xenon relative to krypton and meteorites, with Kr/Xe close to the solar abundance.

The isotopic composition of atmospheric xenon is unique within the solar system. It is isotopically fractionated relative to any known cosmochemical end-member, by about 3.5%.u^−1^ in favour of the heavy isotopes^6^, possibly as a result of the energetic hydrodynamic escape of a hydrogen-rich primordial atmosphere^11^. Such isotope fractionation can be corrected for by assuming mass-dependency, therefore yielding a primordial isotope composition consistent with either a solar or chondritic composition for most isotopes. After correcting for mass fractionation however, the heaviest isotopes ^134^Xe and ^136^Xe of the atmosphere, which were contributed by nucleosynthetic r-process only, are under abundant relative to solar/chondritic compositions. In other words, when chondritic or solar Xe signatures are fractionated by hydrodynamic escape to the extent needed to match the light-isotope ratios of the atmosphere, they contribute more ^136^Xe relative to ^130^Xe than the atmosphere actually contains^11^ (Fig. 1). Subtraction of a putative fission component from air-Xe worsens these discrepancies, implying that pure chondritic or solar Xe sources are firmly ruled out as the only progenitors of terrestrial volatiles. The non-radiogenic terrestrial Xe spectrum (labelled U-Xe) was initially computed from corrections for mass-dependent fractionation and fissiongenic/radiogenic contributions, and multi-dimensional fits to carbonaceous chondrite stepwise heating data^11^. By definition, U-Xe is marked by deficits in ^134^Xe and ^136^Xe relative to common nucleosynthetic reservoirs (solar and chondritic) and, when fractionated by MDF to match air, it falls below the current atmospheric isotope composition at ^129^Xe and ^131–136^Xe, corresponding to later additions of radiogenic (^129^Xe derived from ^129^I) and fissiogenic (^131–136^Xe derived from ^244^Pu) components (Fig. 1). Recently, large depletions in ^134^Xe and ^136^Xe were identified in comet 67 P/Churyumov-Gerasimenko (67 P/C-G)^12^, with the addition of ~22 ± 5% cometary xenon to a chondritic/solar atmosphere being found to match the U-Xe composition^12^ (Fig. 1). This discovery provides a natural mechanism for the origin of U-Xe on Earth, implying the U-Xe signature was not a widespread component in the early solar system, but rather the result of a unique mixing of cometary and chondritic/solar sources on the primitive Earth.

Novel insights into the timing of isotopic fractionation of atmospheric Xe were recently provided by the analysis of ancient atmosphere trapped within fluid inclusions in Archean quartz samples, showing Xe isotope signatures intermediate between U-Xe and the modern atmosphere and pointing towards a global and protracted evolution of atmospheric Xe isotopes^13^. Conversely, Kr isotope signatures in ancient atmosphere samples were consistently found to be indistinguishable from modern composition^13^. To find a higher degree of mass dependent fractionation (MDF, from U-Xe to modern atmosphere) for Xe, despite Kr being a lighter element, is unexpected, suggesting that a Xe-specific process is required to account for atmospheric Xe MDF throughout the Archean^13–16^. Due to the fact that Xe has a low ionization potential relative to the other noble gases, atmospheric Xe could have been readily ionized by enhanced ultraviolet (EUV) radiation^14^. Ionised Xe could then have then been being dragged along open magnetic field lines and lost to space via ionic coupling with escaping H^+^^13,15,16^ or becoming trapped in organic hazes formed within the CH_4_-rich early atmosphere^14^.

Many of the atmosphere’s major constituents, including H, N and Ar are isotopically similar to chondrites. However, Xe in the atmosphere exhibits a high degree of MDF in favour of the heavy isotopes, as well as an underabundance of 10–20 times the value expected given the chondritic abundance pattern of Ne, Ar and Kr on Earth^17–19^ (Fig. 1; Table 1). Both features are referred to as the missing Xe paradox^19^. Untangling the missing Xe problem requires that the ^84^Kr/^132^Xe of the atmosphere and potentially the MDF of atmospheric Xe both be accounted for. The extent of Xe loss to space over the Hadean and Archean eons has not yet been quantified^13,19^. To explain the missing Xe in the atmosphere, it has been proposed that the delivery of solar-like Kr to the atmosphere, with negligible associated Xe addition, following the main stage of atmospheric loss, could potentially resolve the missing Xe (in fact, “extra Kr”)^10^, as well as why Kr isotopes are not fractionated. Pioneering laboratory-based experiments indeed revealed that Kr is trapped more efficiently within amorphous ice than Xe^20^, so a late addition of cometary material with a suspected high ^84^Kr/^132^Xe and solar-like isotopic composition could potentially account for the depletion of Xe on Earth. This could also explain the similar ^84^Kr/^132^Xe abundances on Earth and Mars, which are difficult to reconcile by evoking fractionation processes alone given the substantial differences (e.g., heliocentric distance, mass, timing of formation) between the two planets^21^. Identifying and resolving potential cometary contributions on Earth is vital to understanding how Earth acquired its atmosphere and became hospitable to life.

**Table 1:** ^84^Kr/^132^Xe in different cosmochemical reservoirs of the solar system: comets^31^, chondrites (Q^33^, CC, EC; see Table S1), the subsolar component in EC^53^, solar (range given by^93^, estimated value by^32^), Martian reservoirs (surface^99^ and interior^100^), as well as the present-day terrestrial atmosphere^19^.

	^84^Kr/^132^Xe
Comet 67 P/C-G	4.7 ± 1.1
Q	0.81 ± 0.05
CC	1.20 ± 0.36
EC	1.84 ± 1.14
sub-solar	5.86 ± 0.84
Solar	20–29 (24.4)
Mars atmosphere	20.5 ± 2.5
Mars interior	1.2
Earth atmosphere	27.8

Xenon sequestration in a variety of terrestrial reservoirs (including shales^22^, ice^23^, clathrates^24^, continental crust^25^, as well as Earth’s mantle^26^ and/or core^27^) and preferential retention in the solid Earth relative to other noble gases during degassing have also been proposed to contribute to Xe elemental depletion in the atmosphere. Due to the limited storage capacity of the associated surface reservoirs, the shale, clathrate and ice hypotheses can be ruled out. However, whilst Xe is relatively inert under ambient and neutral conditions, the potential for its enhanced reactivity at high-temperature and high-pressure^28^, and possible incorporation in to mineral phases at depth, is commonly used to argue for Xe to be stored in the Earth’s interior. Here again, although theoretical and experimental investigations suggest possible Xe incorporation into silicate phases found in the Earth’s crust^25^, even the highest measured crustal concentrations of Xe are still three orders of magnitude below that required to account for the missing Xe^29^, therefore excluding the upper continental crust as the main “missing” reservoir. Likewise, Xe abundance in volcanic rocks and xenoliths indicate that the upper mantle reservoir contains 10–100 times less Xe than the present-day atmospheric Xe inventory^30^ (hereafter ATM_Xe_), making it an unlikely resting place for Xe and leaving the deeper mantle and/or core as the only possible sinks. Finally, and perhaps most crucially, explaining the under abundance of atmospheric Xe (relative to Kr and chondritic) by trapping it in silicate reservoirs could only account for abundance ratios and not for Xe isotopic fractionation.

Establishing whether or not the Earth atmosphere is truly deficient in Xe relative to its starting composition requires the composition of the primordial atmosphere to be known. Recently obtained volatile elemental compositions and noble gas isotopic data from Comet 67 P/C-G by the Rosetta spacecraft^12,31^ now offer the potential for cometary and chondritic contributions to the early Earth to be quantified. In this contribution, we present a thought experiment using recently measured volatile elemental ratios and noble gas isotope data from comet 67P/C-G to reconstruct the composition and noble gas signature of the Earth’s ancient atmosphere from simple mixing calculations. First, we use Kr isotopic systematics to elucidate on the nature of the main component (solar vs. chondritic) that was mixed together with comets to form the terrestrial atmosphere precursor. We then determine the cometary contribution required to produce the volatile element compositions (noble gas, water, carbon, nitrogen, halogens) observed in Earth’s modern surface reservoir (ESR: atmosphere, hydrosphere, continental and oceanic crusts). This allows us to simulate the initial atmosphere composition as formed by the mix of comets and chondrites, and compare it with the present-day atmospheric composition in order to test the scenario of Xe loss to space over the Archean eon having partially, or fully, contributed to missing Xe. Finally, correcting the present-day atmosphere for its missing Xe offers the potential for the nature of the chondritic component responsible for most of the volatile element delivery to the ESR to be evaluated, based on its inferred noble gas to water ratio. The main working hypotheses used here to build up this thought experiment and their corresponding implications are presented and discussed in the last section of this manuscript, entitled “Working hypotheses: pros and cons”.

## Identifying the sources of heavy noble gases in the atmosphere

**Figure 2:**
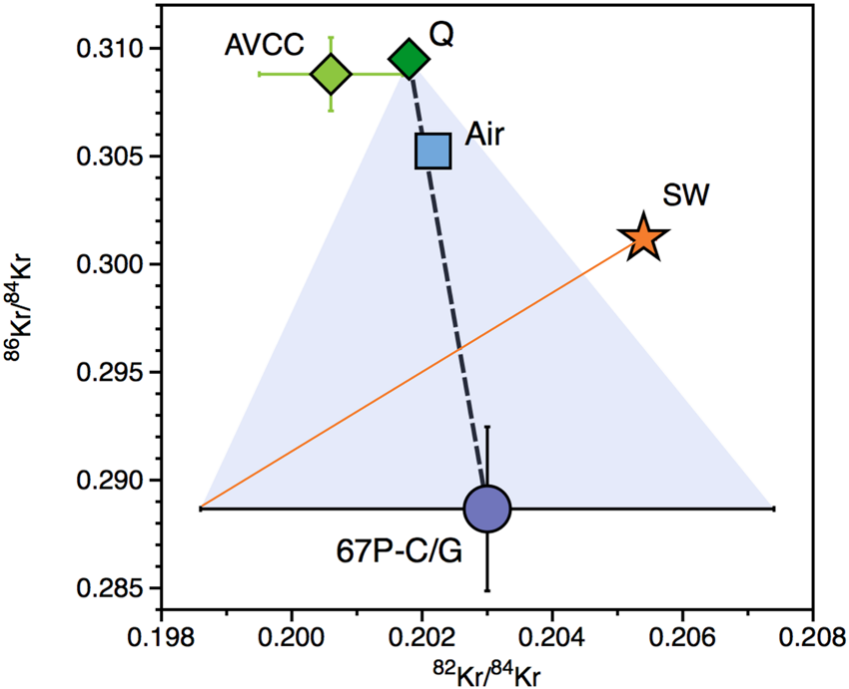
Cometary Kr within the Earth’s atmosphere. The Kr isotopic composition of the atmosphere is shown to lie intermediate between the chondritic Q component^33^ and the composition of comet 67 P/C-G^31^. The unique Kr isotopic composition of the atmosphere could therefore be the result of mixing between chondritic and cometary endmembers (black dashed line). The addition of cometary Kr to an original solar atmosphere^32^ (orange line) fails to reproduce the modern atmosphere. AVCC-Kr^11^ is also displayed. Error bars are 1σ.

Measurements of Xe isotopes within comet 67 P/C-G indicate that the early atmosphere (U-Xe) is the result of mixing between cometary (22%) and chondritic/solar (78%) sources^12^. To address whether cometary noble gases were mixed with solar or chondritic gases to form the atmosphere, we utilise recently determined Kr isotopes from comet 67 P/C-G^31^. Krypton measurements from the Rosetta Orbiter Spectrometer for Ion and Neutral Analysis (ROSINA) aboard the Rosetta spacecraft show that the Kr isotopes from the coma of comet 67 P/C-G are broadly similar to solar, but with depletions in ^83^Kr and ^86^Kr isotopes^31^. Comparing the Kr isotopic composition of comet 67 P/C-G with the two potential atmospheric progenitors, solar^32^ and chondritic (taken to be phase Q^33^), we show that mixing between cometary and Q-Kr can best replicate the composition of the modern atmosphere. Importantly, the atmospheric Kr composition cannot be reproduced by addition of comets to a solar-like signature (Fig. 2), irrespective of the Kr isotopes being considered (Fig. S1). Using Monte Carlo simulations (Supplementary Information), we calculate the amount of cometary Kr addition required to best replicate the modern atmosphere from a chondritic atmosphere as being 21 ± 5%. This suggests that (i) comets may have also contributed significant quantities of Kr to the atmosphere and (ii) cometary noble gases were predominantly mixed with chondritic - and not solar - sources to form the atmosphere^12^. However, as discussed in the “working hypotheses: Pros and Cons” section entitled “, the addition of cometary Kr to a chondritic atmosphere does not succeed in exactly reproducing the ^83^Kr/^86^Kr signature of the atmosphere (Fig. S1), which could be accounted for by the extreme isotopic variability of the comet nucleosynthetic precursors (Fig. S2).

Using the estimate of cometary Kr in the atmosphere we find that the mass of cometary material that accreted to Earth was ~9.8.10^18^ kg (Supplementary Information). This is more than one order of magnitude lower than the mass of comets inferred to have struck the Earth during the purported Late Heavy Bombardment (LHB)^34^, suggesting previous predictions of late cometary accretion may be overestimated. Our estimate however represents a minimum value for the total mass of comets potentially supplied to Earth during main accretion since it does not consider atmospheric loss through impact erosion after the arrival of comets.

## The role of comets in supplying volatiles to Earth

**Figure 3:**
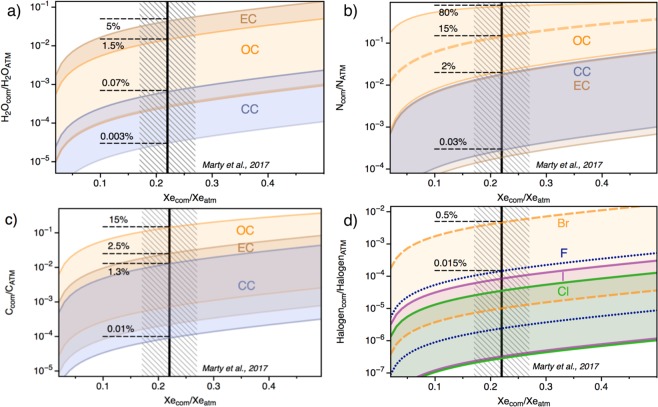
Cometary contribution to terrestrial water (**a**), nitrogen (**b**), carbon (**c**) and halogens (**d**). This is constrained by the 22 ± 5% cometary contribution to terrestrial ^132^Xe^12^, using the ^132^Xe/H_2_O, ^132^Xe/N, ^132^Xe/C, ^132^Xe/F, ^132^Xe/Cl, ^132^Xe/Br and ^132^Xe/I estimated for cometary and chondritic sources (Table 2). While cometary noble gases appear to be extremely enriched in ice, part of halogens present in the comet may reside in the non-volatile fraction of the comet, which possibly escaped measurement from the Rosetta spacecraft. This suggests that estimates for the cometary contribution to terrestrial halogens should be considered as minima. See methods for details on computation.

The amount of other cometary volatiles on Earth may now be calculated from the water, carbon, nitrogen, and halogen to Xe (or Kr) ratios of cometary and chondritic end-members (see methods). Assuming that comet 67 P/C-G is representative of cometary bodies that were scattered throughout the inner solar system (see the “Working hypotheses: Pros and Cons” section), the cometary contribution to terrestrial water, for instance, is computed by combining the cometary Xe/H_2_O^31^ with the requirement to have ~22 ± 5% cometary Xe and Kr contribution to the atmospheric inventory^12^. In this case, the absolute maximum cometary contribution to the ESR budget of water is 5% (Fig. 3). This value relies on the ^132^Xe/H_2_O of the chondritic end-member (Table 2) and is obtained by maximizing the chondritic ^132^Xe/H_2_O, i.e. by using the upper limit given by the dry EC-like component for the chondritic end-member. Increasing the water content of the chondritic-endmember decreases the cometary contribution to terrestrial water (Fig. 3), therefore resulting in cometary contributions to terrestrial water being <0.07% for a CC end-member. Mean cometary contributions to terrestrial water for CC, OC and EC endmembers are 0.016%, 0.15% and 0.2%, respectively. Importantly, these values are in good agreement with the amount of cometary water that would be expected to accrete to Earth (≤2.1.10^18^ kg H_2_O, that is ≤0.15 wt.% of the oceans) from the addition of ~9.8.10^18^ kg of cometary material that is required to account for the cometary Kr contribution in the atmosphere.

**Table 2:** Ι Noble gas, volatile element and halogen composition of chondritic material (CC, OC and EC), comet 67 P/C-G and terrestrial reservoirs (ESR, bulk mantle “BM”) in mol/g.

		Chondrites			Comet	ESR	BM
		CC	OC	EC	67 P/C-G	2.90E^25^g	4.01E^27^g
Noble gases	^36^Ar	(${2.59}_{1.17}^{0.74}$) × 10^−11^	(${3.75}_{7.36}^{2.41}$) × 10^−12^	(${8.32}_{13.1}^{5.56}$) × 10^−12^	(4.31 ± 1.70) × 10^−08^	1.97 × 10^−10^	(7.78 ± 4.29) × 10^−14^
	^84^Kr	(${3.66}_{1.03}^{1.27}$) × 10^−13^	(${7.56}_{4.58}^{2.21}$) × 10^−14^	(${5.93}_{5.94}^{2.86}$) × 10^−14^	(2.52 ± 1,16) × 10^−09^	4.08 × 10^−12^	(1.90 ± 1.10) × 10^−15^
	^132^Xe	(${3.35}_{1.12}^{1.23}$) × 10^−13^	(${6.25}_{2.77}^{1.56}$) × 10^−14^	(${3.64}_{3.50}^{2.29}$) × 10^−14^	(5.34 ± 2.54) × 10^−10^	1.47 × 10^−13^	(1.71 ± 0.45) × 10^−16^
	H_2_O	(${5.71}_{0.57}^{0.56}$) × 10^−03^	(${2.39}_{3.05}^{1.61}$) × 10^−04^	($0.28:2.78$) × 10^−04^	(1.11 ± 0.22) × 10^−02^	3.05 × 10^−03^	(2.00±0.96) × 10^−04^
	δD(‰)	−32 to 92	−120	−130	0 to 2000	0	—
	^14^N	(${5.21}_{1.04}^{1.29}$) × 10^−05^	(${3.57}_{3.75}^{2.73}$) × 10^−07^	(${2.30}_{1.43}^{0.80}$) × 10^−05^	(1.80 ± 1.00) × 10^−03^	1.23 × 10^−05^	(8.98 ± 4.59) × 10^−08^
	δ^15^N(‰)	~ +40	~0	−30 ± 10	840 ± 71	0	—
	^12^C	(${1.67}_{0.46}^{0.41}$) × 10^−03^	(${8.33}_{11.4}^{5,00}$) × 10^−05^	(${3.08}_{0.50}^{0.70}$) × 10^−04^	(3.70 ± 2.80) × 10^−02^	2.66 × 10^−04^	(6.36 ± 2.49) × 10^−05^
Halogens	^35^Cl	(${2.89}_{2.24}^{0.96}$) × 10^−06^	(${1.61}_{3.07}^{1.16}$) × 10^−06^	(${1.68}_{2.15}^{0.92}$) × 10^−06^	(1.55 ± 0.31) × 10^−07^	9.59 × 10^−02^	2.68 × 10^−04^
	^19^F	~2 ×10^−06^	~5 × 10^−07^	(1.05:2.63) × 10^−06^	(7.83 ± 0.62) × 10^−07^	—	—
	^79^Br	(${2.48}_{2.03}^{1.20}$) × 10^−09^	(${7.70}_{26.0}^{6.00}$) × 10^−10^	(${3.98}_{2.94}^{3.19}$) × 10^−09^	(8.60 ± 0.70) × 10^−09^	3.91 × 10^−04^	4.04 × 10^−06^
	^127^I	(${3.15}_{1.14}^{1.28}$) × 10^−10^	(${1.81}_{1.73}^{0.08}$) × 10^−10^	(${4.41}_{0.55}^{2.80}$) × 10^−10^	(4.06 ± 2.23) × 10^−11^	1.23 × 10^−04^	1.01 × 10^−07^

Literature data used to construct this table are provided in Supplementary information. Here, we report values for the median, first (Q1) and third (Q3) quartiles of each data set (${{\rm{Median}}}_{Q3-Median}^{Median-Q1}$, see methods). Concentrations for the ESR and BM are normalized to the mass of the corresponding reservoir, reported in bold beneath the column headers^43^. Given the large uncertainties and limited amount of measurements, the water content in EC is provided as a range of extreme values reported in the literature^79–81^. Note that noble gas elemental ratios may not be derived from this table; we recommend computing average values of compiled ratios (as done here for EC, see supplementary information), rather than ratios of median values.

The same calculation can be performed for nitrogen and carbon, combining the cometary and chondritic ^132^Xe/N and ^132^Xe/C (Table 2) with the 22 ± 5% cometary contribution to terrestrial Xe^12^. The nitrogen depletion in OC relative to EC and CC (Table 2) implies that, if OC were to be the main source of terrestrial volatiles, the cometary contribution to terrestrial nitrogen would be high, on the order of 15%, and up to 80% (Fig. 3). The common high δ^15^N values (840 ± 71)^35^ in cometary matter exclude significant cometary contribution to the terrestrial N inventory (δ^15^N = 0‰). High cometary contributions to the terrestrial budget of N in the case of a binary mixture between OC and comets therefore potentially precludes OC as the main source for chondritic volatiles on Earth’s surface. Conversely, CC- and EC-like end-members require the cometary contribution to terrestrial nitrogen to be ≤2% (Fig. 3). Although the quantities of major volatile species delivered to Earth from comets are minor compared to chondrites, they may still have had a ubstantial impact on the geochemical signature of the Earth’s volatiles, given the extreme differences in isotopic signatures between these two accretionary reservoirs. For instance, the addition of a few percent cometary N, enriched in heavy ^15^N^35^ could substantially raise the δ^15^N of the atmosphere from primitive negative values found in the mantle (δ^15^N = −25‰)^36^ towards the current atmospheric value (Fig. S3)

Mixing comets with a CC-like endmember requires the cometary carbon contribution to the ESR to be low (mean contribution of 0.6%; Fig. 3). Mixing with OC or EC endmembers on the other hand leaves the possibility for comets to have contributed up to 15% of terrestrial carbon. Our estimates of the maximum amount of cometary organic materials supplied to Earth after the Moon forming impact (<4 × 10^21^g, see methods) indicate that comets potentially provided up to 2000 times the present day mass of the biosphere (∼2 × 10^18^g). Interestingly, CC materials constituting up to 20wt.% of the late veneer^37^ could have contributed up to 1 × 10^23^g of organic matter, in agreement with the fact that comets could only contribute a few wt.% at maximum to the budget of Earth surface’s carbon in the case of significant contribution from CC-like bodies (Fig. 3). In the case of a limited contribution from CC-like material in the ESR, there is a possibility for comets to have contributed significantly (several tens of wt.%) to the carbon budget in the ESR (Fig. 3). In addition, comets may be enriched in prebiotic molecules relative to chondrites, with potentially up to ~500 μg/g glycine in comets^38^ compared to ~2.5 μg/g in CM chondrites^39^. Based on these estimates, we calculate that up to 5 × 10^18^g of cometary glycine could therefore have been supplied to early Earth, which is significantly higher than the maximum estimated contribution from CC as part of the late veneer (3.6 × 10^17^g of glycine). However, the preservation rate of extraterrestrial, biologically relevant molecules upon delivery to early Earth depends on the physical properties of the impactor (size, density, porosity, speed, trajectory) and the atmosphere (density, height, composition; Fig. S4)^40^. The low density and high porosity of comet 67 P/C-G^41^ could offer the potential for efficient aerobraking of small cometary impactors and early fragmentation upon atmospheric entry, therefore limiting pyrolysis (and thus favouring potential preservation) of organic molecules (Fig. S4). Yet, a genetic link between extraterrestrial organic materials (cometary or chondritic) and the emergence of life on Earth remains poorly understood.

Finally, using the halogen concentrations in the coma of Comet 67 P/C-G^42^, we calculate that comets contributed very limited amounts (<0.5%) to the budget of terrestrial halogens (Fig. 3), in line with the elemental and isotopic ratios of heavy halogens being chondritic on Earth^43^. The terrestrial Br/Cl ((3.0 ± 0.2) × 10^–3^) is indeed indistinguishable from the chondritic ratio ((2.6 ± 0.8) × 10^−3^)^43^, but significantly lower than the cometary value (in the range 0.02–0.2, with a mean value of 0.08)^42^. However, part of halogens present in the comet may reside in the non-volatile fraction of the comet, including organic phases, which was not measured by the Rosina instrument on-board the Rosetta spacecraft. For instance, the iodine content of comet 67 P/C-G ice is too low to account for the ^129^Xe excess observed in the ice of comet 67 P/C-G^12^. Using this ^129^Xe excess to derive a minimal amount of additional cometary iodine, assuming a ^129^I/^127^I ratio at the moment of the solar system formation of ~1 × 10^−4^ ^44^, requires the comets to have an unrealistic bulk ^127^I concentration around 230 ppm, suggesting the large ^129^Xe monoisotopic excess observed in the comet originate from (i) a specific nucleosynthetic process preferentially producing ^129^Xe or (ii) decay of ^129^I in the ambient gas or dust before solar system formation^12^.

**Figure 4:**
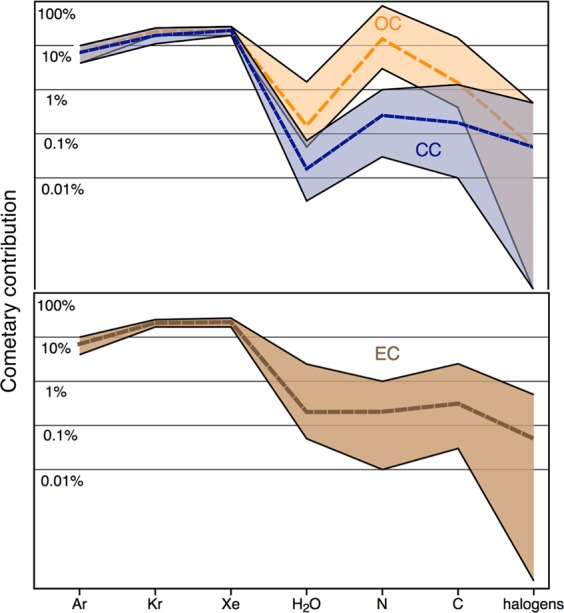
Cometary contribution to the ESR inventory of noble gases, water, nitrogen, carbon and halogens. Medians of distribution are displayed as dashed lines. Comets likely contributed negligibly to all the aforementioned volatile species, except for the heavy noble gases (~20%). High cometary contribution to terrestrial nitrogen, as required in the case of a binary mixture between OC and comets, preclude OC-like materials as the main source of chondritic volatiles in the atmosphere.

Our model predicts comets to have contributed negligibly to all the aforementioned volatile species, except for the heavy noble gases, whose concentrations are high in cometary ice^31^ (Fig. 4). Importantly, the timing of chondritic and cometary deliveries with respect to the Moon-forming giant impact (occurring ≥40 Myr after solar system formation^45^) is crucial to address their potential for having supplied organic materials that contributed to the emergence of life. Due to the fact that comets are extremely rich in organic materials and have high eccentricities and hyperbolic trajectories, they are compelling candidates for having seeded early Earth with biologically relevant molecules. The contrasted heavy noble gas signatures in the Earth’s mantle (chondritic^46,47^) and atmosphere (mixture of 20% cometary plus 80% chondritic^12^) may be used to argue for a late cometary bombardment of the Earth, possibly related to the giant planet instability about 3.9 Gy ago (referred to as the Nice model)^18,48^. The latter would notably have triggered the dispersal of the trans-Neptunian disk and of the asteroid belt, leading to a heavy bombardment of all terrestrial planets. One promising avenue of investigation to further constrain the timing of cometary supplies to the Earth-Moon system is to determine whether or not cometary volatiles are preserved on the present day Moon surface^49–51^.

## Constraining the extent of “Xe loss”

**Figure 5:**
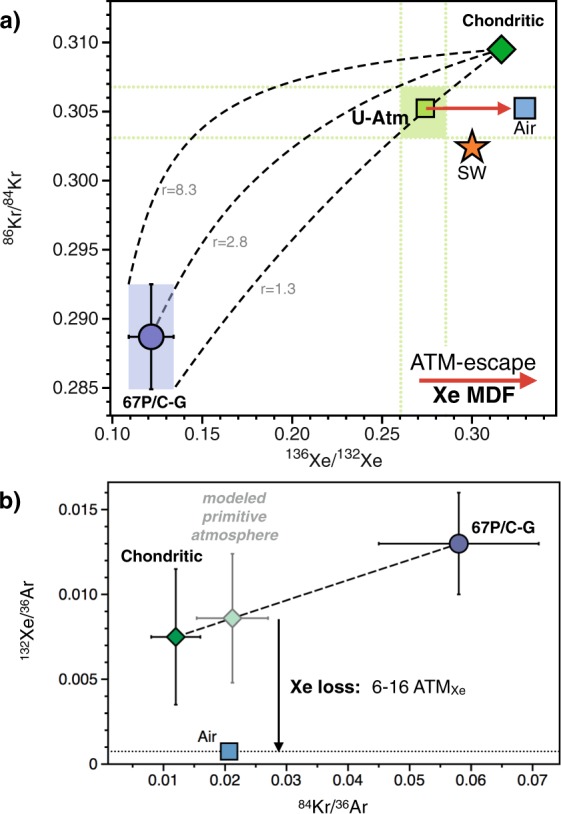
Origin and evolution of heavy noble gases in the terrestrial atmosphere from isotopic (**a**) and elemental (**b**) constraints. (**a**) Dashed curves represent mixing lines between comet 67P/C-G and the chondritic Q component with ratios (r) equalling 8.3, 2.8 and 1.3, defined by the maximum, mean and minimum values for the ^84^Kr/^132^Xe_comets_ / ^84^Kr/^132^Xe_chondrites_, respectively. Whereas the isotopic composition of the chondritic end-member is taken as Q^33^, the considered range of elemental compositions corresponds to that of EC, which spans all chondritic ratios reported in Table 1. Horizontal and vertical doted lines show ranges of values corresponding to the 21 ± 5% cometary Kr and 22 ± 5% cometary Xe^12^ required to form the primitive atmosphere. Reconciling elemental and isotopic constraints requires the Kr and Xe isotope compositions of the primitive atmosphere to be indistinguishable from modern atmospheric Kr and U-Xe, respectively (U-Atm). Subsequent fractionation of Xe isotopes toward higher ^136^Xe/^132^Xe would account for the present day composition of air, with limited contribution from fission-derived Xe. The Xe-specific process driving atmospheric Xe MDF in favour of the heavy isotopes over the Archean^13,16^ could - at least partially - account for the Earth’s missing Xe, with negligible loss (if any) of atmospheric Kr through time. (**b**) Modelling the primitive atmosphere by mixing 80% chondritic and 20% cometary noble gases^31^ requires the subsequent loss of 6 to 16 times the present day inventory of atmospheric Xe to account for the modern composition of the terrestrial atmosphere.

**Figure 6:**
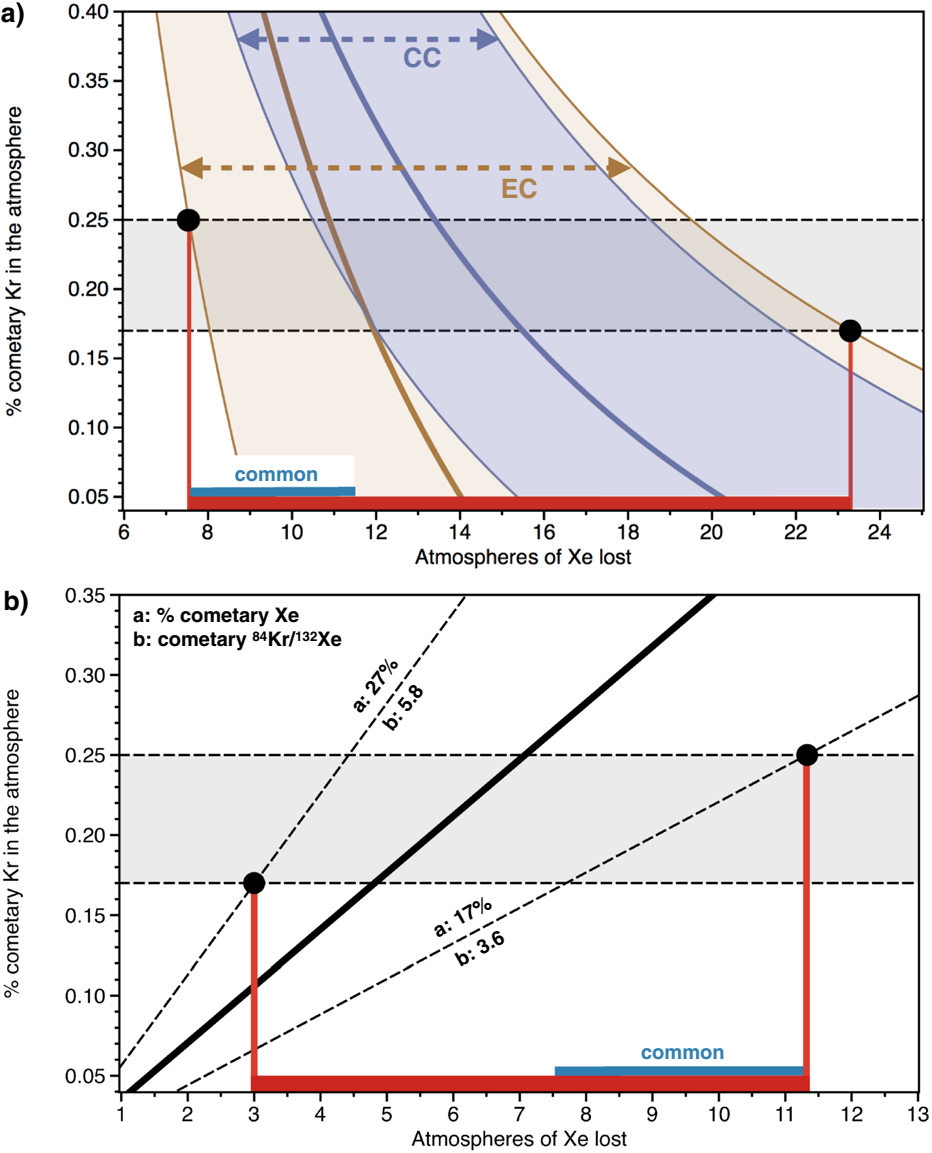
Constraining the extent of the Earth atmosphere missing Xe (**a**) Discrepancy between the ^84^Kr/^132^Xe of the initial atmosphere, as formed by the mix of comets and chondrites (Table 1), and its present-day ^84^Kr/^132^Xe permits the amount of lost atmospheric Xe to be derived as a function of the ^84^Kr/^132^Xe of the chondritic end-member (7.5–23 Xe atmospheric masses lost). The thick brown and blue curved lines represent the medians of distribution for mixing with EC and CC, respectively. **(b)** Discrepancy between the ^84^Kr/^132^Xe of comet 67 P/C-G (4.7 ± 1.1)^31^ and the calculated cometary Kr and Xe contributions (~20%) enables the extent of atmospheric Xe loss to be estimated (3–11.5 Xe atmospheric masses lost). The “common” range: the only possibility for the extent of Xe loss from the atmosphere is at the intersection between 7.5–23 ATM_Xe_ and 3–11.5 ATM_Xe_, i.e. ~8 to 12 ATM_Xe_ being missing from the present-day inventory. See methods for details on computation.

The depletion of Xe (missing Xe) in the atmosphere from the expected chondritic abundance pattern (Fig. 1) remains an outstanding problem regarding the origin of the atmosphere. Mixing Kr and Xe between cometary and chondritic sources by taking into account the elemental ratios of each endmember is able to directly reproduce the isotopic composition of U-Xe, the modern isotopic composition of terrestrial atmosphere Kr and the ^84^Kr/^36^Ar ratio of modern atmosphere (Fig. 5). However, accounting for the present day Xe isotope composition and ^132^Xe/^36^Ar of the atmosphere requires atmospheric Xe to have been both fractionated in favour of the heavy isotopes, and to have lost 6–16 times the present-day atmospheric inventory of Xe (Fig. 5). Here, we combine two different approaches to set constraints on the extent of Xe specific loss to space after completion of the primitive atmosphere. The first method (A) computes the difference between the ^84^Kr/^132^Xe of the initial atmosphere, as formed by the mix of comets and chondrites, and the present-day atmospheric ^84^Kr/^132^Xe, therefore requiring the ^84^Kr/^132^Xe of the chondritic component in the atmosphere to be defined. The second method (B) relies only on the apparent level of missing cometary Xe, without the need to define a chondritic endmember. Details regarding these two methods are provided in the Methods section.

(A) The terrestrial atmosphere, taken as a mix of comets (~20%) and chondrites (~80%) for ^84^Kr and ^132^Xe would result in a ^84^Kr/^132^Xe ~3, which is significantly lower than the present-day atmospheric value (27.8, respectively; Table 1). Increasing the early atmospheric ^84^Kr/^132^Xe from ~3 to 27.8 requires a mechanism to account for the specific loss of atmospheric Xe. We show that, to form the modern atmosphere from the original mix of chondritic and cometary noble gases, between 7.5–23 ATM_Xe_ must be lost (Fig. 6a), in agreement with previous estimates for the extent of Earth’s missing Xe (10–20 masses of ATM_Xe_)^19^. This computation relies on the ^84^Kr/^132^Xe of the chondritic end-member (Table 2), which has long been recognized to be ~1.3 ± 0.3 for bulk CC and OC^52^ (see value of 1.20 ± 0.36 from our own compilation, Table S1) and to be more variable for EC (see Table S1).

(B) The observation that the isotopic composition of atmospheric Kr has remained constant since the early Archean^13^ suggests that the total inventory of Kr in the ESR might have been little affected since the last major episode of impact erosion of the atmosphere, considered to be the Moon forming event. From the amount of cometary Kr residing in the ESR (21 ± 5%), we utilise the cometary ^84^Kr/^132^Xe ratio^31^ to determine theoretical amounts of cometary Xe that should be expected in the ESR. Comparing these theoretical values with the previous estimates of cometary Xe in the ESR, i.e. 22 ± 5% of the total inventory, yields that 3–11.5 ATM_Xe_ are missing from the ESR (Fig. 6b).

The ^84^Kr/^132^Xe ratio in comets (=4.7 ± 1.1) as measured in the coma of Comet 67P/C-G^31^ indicates that comets may have chondritic-like noble gas elemental ratios, and not solar ones (Table 1). In order to have the same cometary contribution for both Kr and Xe (around 20%), mass balance considerations indeed require that cometary and chondritic end members that formed the Earth had similar ^84^Kr/^132^Xe ratios. Taking into consideration the range of ^84^Kr/^132^Xe values measured in Comet 67 P/C-G and the uncertainties associated with the isotopic mixing constraints (21 ± 5% and 22 ± 5% cometary Kr and Xe, respectively) necessitates the chondritic ^84^Kr/^132^Xe component to be ≥2. Enstatite chondrites have abundant sub-solar noble gas component (^84^Kr/^132^Xe = 5.86 ± 0.84)^53^, causing their bulk ^84^Kr/^132^Xe to be higher and more variable than other chondritic types (Table 1). Whilst accounting for the isotopic ratios of Kr released from ECs does not necessarily require a solar Kr contribution^53^, Xe data for some subsolar gas-carrying ECs suggest the presence of SW-Xe. Okazaki *et al*. (2010) acknowledged that the Xe composition of subsolar gas may also be identical to that of Q gas. Hence, although the subsolar component in ECs may not constitute an isotopic component, it has a significant effect on bulk noble gas elemental ratios, specifically in raising the ^84^Kr/^132^Xe to higher values. Taken together, these data suggest that a source of solar-wind irradiated materials akin to that which accreted to form the EC parent body (or angrites, as seen from the analysis of D’Orbigny glass samples^54^) might be required to form the noble gas composition of the primitive atmosphere. Independent estimates of Xe loss assuming an EC-like chondritic endmember result in a common range of possible values, at the intersection between 3–11.5 ATM_Xe_ and 7.5–23 ATM_Xe_, i.e. ~8 to 12 atmosphere masses of Xe being lost from the present-day inventory (see “common” range, Fig. 6).

The non-solar ^84^Kr/^132^Xe ratio for the comet 67 P/C-G^31^ refutes the idea that the two-step atmospheric formation model of fractionated solar plus late cometary addition^10^ was responsible for the origin and evolution of the atmospheric noble gas composition. The present finding that up to 12 atmospheres of Xe could have been lost from the atmosphere since the Hadean eon potentially resolves the long-standing mystery of Earth’s “missing xenon”, which turns out to be predominantly “lost Xe”. Assuming a Rayleigh-type distillation during the loss of 8–12 ATM_Xe_ to space requires the instantaneous isotopic fractionation α to be high, in the range 15–18‰u^−1^ ^13^. To date, significant isotopic fractionation (10 ± 4‰u^−1^) has only been achieved under ionizing conditions^55,56^, as suggested to occur in models of Xe loss to space and trapping in organic hazes^14^. The preferential incorporation of heavier Xe isotopes into archean organic hazes would have indeed left the atmosphere enriched in light Xe. Any subsequent atmospheric loss of this light Xe could have ultimately resulted in the heavy Xe-enriched atmosphere we see today^14^. High α values up to 18‰u^−1^ might therefore result from the combination of isotopic effects from both escaping to space, and trapping into organic hazes.

Martian meteorites suggest that escape of atmospheric Xe terminated much earlier (4.2–4.3 Gyr ago) on Mars relative to Earth^57^. The early formation of Mars (≤2.7 Myr after CAI formation)^58^ may imply that the EUV flux responsible for Xe ionization and escape on Earth was much stronger in the early Martian atmosphere. In combination with the lower escape velocities expected on Mars, one can predict early and extensive Xe escape from the Martian atmosphere. Interestingly, the presence of Martian methane^59^ indicates that trapping and fractionating ionized Xe in organic hazes might also have been a viable process for driving atmospheric Xe evolution on Mars. Given the differences between terrestrial planets, the atmospheres of Venus, Earth and Mars probably experienced contrasted evolutions. Analysing the Venusian atmosphere for its Kr and Xe composition therefore has the potential to discriminate between models of Xe fractionation on common planetary precursors^58^, versus on planets themselves, as suggested by this study.

The possibility for some Xe to have partially remained sequestrated in the solid Earth under the form of stable chemical compounds, and for this mechanism to have contributed to the missing Xe paradox, cannot be discarded. However, we note that the potential to have sequestrated Xe in a given reservoir does not solve the missing Xe problem if there is a chance for Ar and Kr to be likewise sequestered - as seems to be the case^60^. To this extent, the mechanism accounting for missing Xe is required, not only to apply to Xe, but to be Xe specific. Because of their high density, solid Xe particles could preferentially segregate in the Earth’s deeper regions^61^. However, the potential for large enough solid Xe inclusions to form and be transported into the core through gravitational segregation is highly questionable given the extremely low Xe abundance of the mantle. At present, less than 10% of the total inventory of heavy noble gases in the bulk silicate Earth resides in the mantle (Table 2). For the missing Xe to reside in the core would require the latter to store 10 to 20 ATM_Xe_, i.e. more than 100 to 200 times more Xe than the total inventory of the mantle. Although Xe oxides are stable in the Xe–O system at pressures and temperatures of the Earth’s lower mantle, neither Xe silicates, which would naturally decompose, nor oxides, which would be reduced by metallic iron, can exist in its strongly reducing environment^62^. Theory and experiments have also demonstrated that Xe and Fe do not react at pressures up to 155 GPa^ 63,64^, therefore arguing against the likelihood of Xe partitioning into core-forming metal. Recently, Zhu *et al*.^27^ showed that stable XeNi_3_ and XeFe_3_ would emerge at 200 GPa and 250 GPa, respectively. However, difficulties arise in finding a mechanism that can trap Xe at low pressure and deliver it to a depth where such stable Xe-bearing phases would emerge. In other terms, stable Xe–Fe and/or Xe-Ni compounds would be unstable until they reach core pressures, and so the process by which iron and/or nickel could have dragged Xe into the core are not well understood. Importantly, the high pressures required for potentially trapping some Xe in the deep mantle phases on Earth could not be achieved on Mars, where the maximum pressure in the core only reaches 50 GPa ^65^, further suggesting that this mechanism is unlikely to have resulted in the missing Xe observed for Mars and Earth. Interestingly, the much higher solubility of Ar in MgSiO_3_ perovskite relative to Xe led Shcheka and Keppler (2012)^66^ to suggest that, instead of having been preferentially retained in the solid Earth, Xe might have been depleted very early in the Earth’s lower mantle relative to lighter noble gases during crystallization of perovskite from a magma ocean. The purported Xe-rich primordial atmosphere could then have been lost to space, before degassing of the lower mantle replenished Ar and Kr, but not Xe, into the atmosphere. Importantly, as it is the case for all of the aforementioned models, this scenario cannot account for the observed isotopic evolution of atmospheric Xe over the Archean eon^13^. Xenon ions escape in a photo-ionized hydrogen wind, possible in the absence of a geomagnetic field or along polar magnetic field lines that open into interplanetary space^16^, is so far the only viable mechanism accounting for both the isotopic composition and elemental depletion of Xe in the terrestrial atmosphere.

## On the nature of the chondritic component in the atmosphere

**Figure 7:**
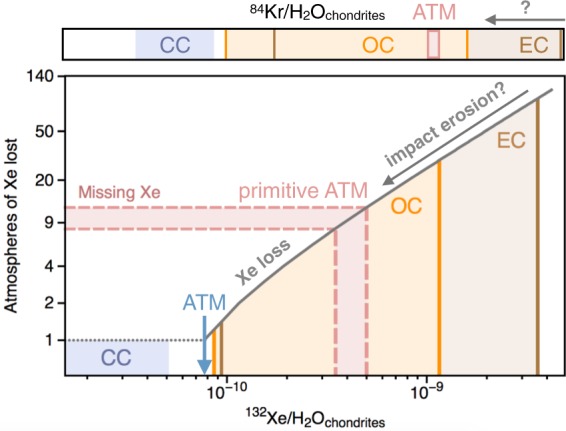
Origin and evolution of heavy noble gases in the terrestrial atmosphere as constrained by the extent of missing Xe. The present-day atmosphere is corrected for 8–12 atmospheric masses of Xe loss, therefore allowing its primitive ^132^Xe/H_2_O to be estimated. While impact erosion of the atmosphere could have driven preferential loss of noble gases relative to water during accretion (i.e. decreasing ^132^Xe/H_2_O)^1^, a preferential loss of water relative to noble gases during accretion (i.e. increasing ^132^Xe/H_2_O) is unlikely. The finding that the ^132^Xe/H_2_O of the proto-Earth may have been toward the end-member of EC, which were progressively supplied throughout the Earth’s accretion history, suggests that the majority of the Earth’ surface volatiles may have been delivered during the main phases of terrestrial accretion. This is in line with the ^84^Kr/H_2_O of the ESR being similar to EC, but distinct from CC.

Variations in the noble gas to water ratio over chondritic types are extremely large (several orders of magnitude difference between EC and CC). Although elemental ratios in meteorites may change with time and during processes involving thermal metamorphism and/or aqueous alteration^33^, differences in the noble gas to water ratio between CC and EC/OC are primarily controlled by the amount of water within the respective chondritic types, reflecting the heliocentric distance at which the parent bodies accreted^5^. Low ^84^Kr/H_2_O ratios (~6.2 × 10^−11^) are therefore characteristic of CC, whilst ^84^Kr/H_2_O ratios over ~5.3 × 10^–10^ are representative of drier materials akin to EC and OC (Table 2). Although the present-day ^84^Kr/H_2_O ratio of the ESR appears to plot in the range of OC and EC, the ^132^Xe/H_2_O of the ESR is close to (but still higher than) that of CC (Fig. 7; Table 2). This is likely due to the fact that the present-day ^132^Xe/H_2_O of the ESR needs to be corrected for Xe loss to represent the ^132^Xe/H_2_O of the primitive ESR. By correcting the present-day inventory of the ESR for Xe loss, we find that the original ^132^Xe/H_2_O of the primitive Earth also matches the composition of chondritic materials with ^132^Xe/H_2_O similar to OC and EC (Table 2; Fig. 7). In order to compare the noble gas to water ratio of the primitive Earth as formed by mixtures of comets and chondrites with that of the present day ESR, we need to now estimate how planetary processes would have affected the noble gas to water ratio of the ESR over geological periods of time.

The atmospheric mass and composition of the early Earth likely resulted from an interplay of impact erosion, volatile element dissolution in the magma ocean, outgassing from the solid Earth, and late deliveries^1^. Although preferential dissolution of water into the magma ocean would have raised the noble gas to water ratio of the Earth’s surface, the vast majority of water dissolved within the magma ocean is expected to have been expelled back to the atmosphere during rapid bottom-up magma ocean solidification^3,67,68^, leaving only a small fraction of the original volatiles to be released into the atmosphere through later volcanism. The final amount of water in the atmosphere should therefore be close to the initial budget of the atmosphere and magma ocean^3^. After magma ocean solidification and cooling, a water ocean is expected to rapidly form on the surface of the proto-Earth^68^. The presence of an ocean at the surface of a protoplanet during subsequent impacts has been shown to extensively enhance atmospheric loss to space^2^. Whilst impact-driven loss may not elementally or isotopically fractionate the noble gases as it involves bulk ejection of the atmosphere, it can efficiently contribute to their overall depletion with respect to water^1^. Interestingly, the proto-Earth probably experienced multiple events of giant impact leading to sequential episodes of magma ocean outgassing and rapid formation of proto-oceans^69^, so impact erosion of the atmosphere likely played a key role in altering the Earth’s atmospheric mass and composition^2^, including the noble gas to water ratio of the ESR.

Partial loss of water from the ESR by hydrodynamic escape and/or subduction of seawater to the mantle could also have modified the noble gas to water ratio of the ESR over geological periods of time. The loss of 1/3 of a global ocean unit by hydrodynamic escape of H_2_ has been suggested to account for the shift in f(O_2_) state of the upper mantle from the very low oxidation state equivalent to primitive differentiated bodies, to its present oxidized state^70^. Models of Xe ions escape in photo-ionized hydrogen winds also predict that the extent of hydrogen loss required to account for atmospheric Xe evolution could be one order of magnitude less than the Mass of the present day Terrestrial Oceans^16^ (1MTO = 1.38 × 10^24^g of H_2_O). Parai & Mukhopadhyay (2018)^71^ recently used the isotopic composition of Xe in the geological record (ancient atmosphere and mantle) to constrain the timing of onset and extent of volatile elements recycling into the solid Earth. They show that downwellings before 2.5 Ga were arguably dry, with the median H_2_O concentration in downwellings at 3 Ga being ~0.61 p.p.m. H_2_O, compared to modern-day slabs subducting beyond depths of magma generation (~400–1,000 p.p.m. H_2_O^72^). According to continental freeboard studies, sea level changes since the Archean were limited to less than 500m^73^. The total mass of water within the ESR is therefore thought to have been established early and to have remained roughly constant over geological periods of time, potentially since the conclusion of the Earth’s last main accretionary stage, with the net flux of water to the surface being balanced by the return through subduction^74^. The initial mass of the early oceans, as determined by mass balance calculations, would have been 20wt.% greater than at present^75^, suggesting that variations in the budget of water in the ESR would have been limited since formation of the primitive ocean, after magma ocean solidification. The noble gas to water ratio of the ESR could therefore have been established early during Earth’s history, with impact-driven loss of the early atmosphere constituting the main process in driving noble gas to water fractionation of the ESR with respect to its precursor material composition. In this case, the noble gas to water ratio of the ESR should be taken to reflect a minimal value for the noble gas to water ratio of the building blocks it accreted from.

**Figure 8:**
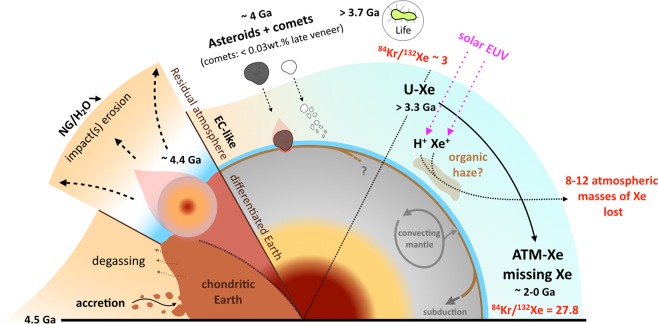
Schematic view of the origin and evolution of heavy noble gases in the atmosphere. Heavy noble gases are chondritic in the solid Earth^46,47^, in line with the Earth being dominantly composed of chondritic accreting blocks. The Earth’s early atmosphere has a complex history of impact erosion, outgassing from the solid Earth and late supplies by asteroidal and cometary bombardments. The addition of ~22% cometary heavy noble gases to a Q-like^33^ atmosphere is able to resolve the origin of U-Xe^12^ and atmospheric Kr. Subsequent and protracted fractionation of atmospheric Xe is thought to have been driven by Xe escape to space with 8–12 atmospheric masses of Xe being lost over the Archean eon^13^.

The possibility to have had even drier precursors (i.e., with higher noble gas to water ratios) and a preferential loss of Kr andXe relative to H_2_O during impact events leaves the potential for Earth to have predominantly acquired its volatile elements from asteroids with water contents akin to EC (Figs. 7–8). Taking the maximum extent of previously determined missing Xe to reach 20*ATM_Xe_^19^ would further increase the inferred ^132^Xe/H_2_O of the primitive Earth surface (Fig. 7), and strengthen our interpretation. Finally, estimates of water contents in the bulk mantle range from 1 MTO^76^ up to 12 MTO^6^, with a mean value of around 2 to 3 MTO potentially residing in the solid Earth^77^. When considering an extreme scenario where all the water now potentially residing in the Earth’s mantle originated from the surface would lower the ^84^Kr/H_2_O of the primitive Earth down to 3.2.10^−10^ and 1.4.10^−10^ for 5 MTO and 12 MTO, respectively, yet still remaining above the maximum estimated ^84^Kr/H_2_O of CC (9.5.10^−11^).

Whilst it is commonly thought that planets in the inner solar system first grew dry, with water and atmophile elements being subsequently contributed by volatile-rich materials originating from larger heliocentric distances^6,78,79^, our results suggest that CC may not have been the primary source of volatiles in the ESR. This is in line with the predominantly inner solar system origin of the latest stages of Earth’s accretion^8,9^. Bulk water concentrations for EC range from <0.05%^80^ to ~0.5%^81^, indicating that the 100% EC-like phase of the final 40% of terrestrial accretion^9^ can provide between 0.8 and 8 MTO), with the late veneer potentially supplying another 0.7 MTO^37^. This suggests that the bulk of Earth’s water could have accreted with its main building blocks, before the giant impact^37^, and that Earth’s volatiles potentially originated from drier precursors than previously thought. However, estimates of water contents in EC are highly variable^81^, with the ever-present issue of air contamination correction. Firmly establishing whether or not the latest stages of Earth’s main accretion could have contributed sufficient water to the ESR will require additional, more precise measurements of water in EC. The main conclusions of this contribution regarding the origin and evolution of volatile elements - especially heavy noble gases - in the ESR are reported in Fig. 8.

## Working hypotheses: pros and cons

The present paper uses previously published data on the noble gas isotopic composition and volatile element content of cometary and chondritic materials to infer on the origin and composition of the primitive ESR. In line with results from previous studies^18,79^, we predict comets to have contributed negligibly to the terrestrial volatile budget, except for the heavy noble gases. This work includes some assumptions that we outline in detail below, regarding (a) the relationship between the isotopic signature of comet 67P/C-G and other components in the solar system, and its representativeness for the bulk cometary reservoir, (b) the composition of cometary and chondritic end-members for mixing calculations, and (c) the effect of planetary processes (e.g., subduction, impacts, hydrodynamic escape) on the global noble gas to water ratio of the ESR.

(a) The European Space Agency Rosetta mission to comet 67 P/C-G represents the first detailed *in situ* measurements including trace elements such as noble gas isotopes of a cometary body in the solar system. Nucleosynthetic differences between distinct solar system reservoirs, such as observed for Ti, Sr, Ca, Cr, Ni, Zr, Mo, Ru, Ba, Nd, Sm, Hf, W, and Os isotopes between CC (from the outer solar system) and EC/OC (from the inner solar system)^82^, indicate that nucleosynthetic heterogeneities may develop over large spatially distinct reservoirs in the solar system. The discovery of molecular O^83^ and S_2_^84^ in the coma of comet 67P/C-G, as well as small deviations of ^12^C/^13^C in CO and CO_2_ relative to solar composition^85^, suggests it formed at least in part from icy grains originating from the interstellar medium. A possibility is that the noble gas signature of comet 67P/C-G represents the composition of the outer solar system/interstellar reservoir from which comets originally formed^12^ and therefore, the noble gas signature of comets having accreted from the same region as comet 67P/C-G should reflect a similar nucleosynthetic mixture. In this framework, it is however surprising that no evidence for a cometary noble gas contribution to the CC reservoir (especially Xe) could be found so far^86^.

The Kr and Xe signatures measured in comet 67P/C-G define a nucleosynthetic component that is unique within our solar system, with notable depletions in ^83^Kr, ^86^Kr, ^134^Xe and ^136^Xe relative to the “normal” (N) composition^12,31^. The Kr isotopic signature of comet 67 P/C-G would be best accounted for by a mix of presolar N-Kr plus 2–5% G-Kr^31,87^ (Fig. S6), with additions of G-Kr being invoked to account for the deficit in ^83^Kr/^84^Kr relative to N-Kr. However, the G-Kr nucleosynthetic component shows such a huge isotopic variability relative to N-Kr^87,88^ that the final Kr isotopic composition of any cometary material formed by mixing G and N components would be highly sensitive to the extent of G-Kr contribution (Fig. S2). Although we consider the presolar Kr signature of comet 67P/C-G to be representative of Jupiter family comets (JFC) in general, cometary materials with small variations in the amount of G-Kr could have brought cometary Kr to Earth with a slightly different isotopic signature than that measured in comet 67P/C-G (Fig. S2). This may in part explain why the addition of comet 67P/C-G to a chondritic atmosphere does not succeed in exactly reproducing the ^83^Kr/^86^Kr signature of the atmosphere (Fig. S1).

**Figure 9:**
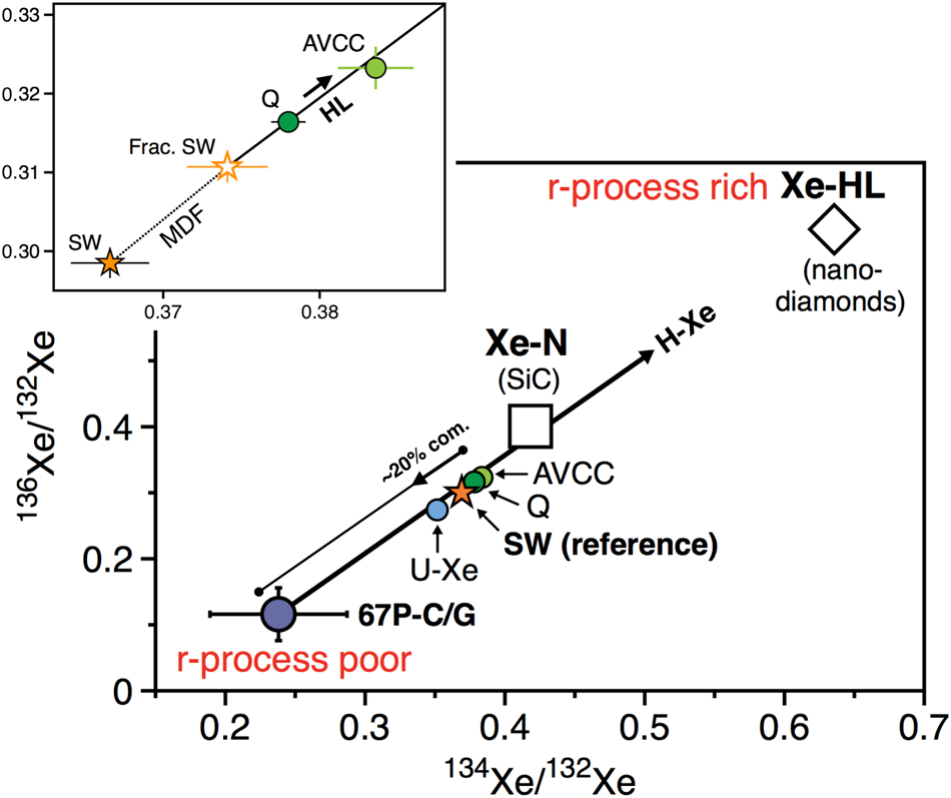
Three-isotope diagram of Xe isotopes showing the relationships between planetary and cosmochemical reservoirs. The solar composition (SW)^32^ is intermediate between, on one side, the r-process depleted cometary signature^12^, and, on the other side, the Xe-HL and Xe-N components carried out by presolar nanodiamonds and SiC, respectively^87,88^. The relationship between SW, Q and AVCC is shown in the upper left panel. The AVCC signature represents a slightly higher contribution of Xe-HL from fractionated SW-Xe, relative to Q-Xe^86^, which was itself produced by 98.5% SW-Xe that experienced linear mass dependent fractionation of 10.2 u^-1^ favouring the heavy isotopes, and mixing with 1.5% Xe-HL^88^. At last, we show the U-Xe composition^11^, accounted for by mixing ~20% cometary Xe with ~80% chondritic Xe^12^. In this plot, we note that the MDF line (dotted line) is interestingly very close to general mixing trends.

The Xe isotope composition of comet 67P/C-G is marked by a deficit in isotopes that are only produced by r-process (i.e., ^134^Xe and ^136^Xe) relative to common nucleosynthetic reservoirs (solar and chondritic; Fig. 9). Conversely, the Xe-HL and Xe-N components carried by presolar nanodiamonds and SiC, respectively^88^, are characterized by higher ^134^Xe/^132^Xe and ^136^Xe/^132^Xe relative to the solar composition (Fig. 9). Importantly, all Xe isotope cosmochemical reservoirs within the solar system are related by MDF and mixing relationships (Fig. 9). The isotopic composition of Xe in chondrites and solar wind could potentially have derived originally from cometary Xe through the addition of r-process rich material^11^ (e.g. ~3.2% of Xe-H, with the Xe-H composition from^89^). Comets may therefore constitute our best estimate for the composition of the primitive solar system. This scenario requires that p-process derived (Xe-L, “light”) and r-process derived (Xe-H, “heavy”) fractions of Xe-HL be separated from one another, which, although expected from an astrophysical point of view, is at odds with their constant abundance ratio in presolar nanodiamonds found in meteorites^11,86,88^. If Xe-L and Xe-H are considered to be inseparable from one another, then this would require cometary Xe to also have had a deficit in p-process derived isotopes relative to the solar composition, which could only be compensated for by a subsequent addition of pure p-process Xe^90^ - in itself inconsistent with the initial hypothesis of Xe-L and Xe-H being inseparable. Interestingly, the relative r-process deficit in cometary material could be a widespread feature of the bulk cometary reservoir. For instance, metal-rich carbonaceous chondrites thought to have accreted in the outermost regions of the PSN, beyond the orbit of Neptune, are thought to have accreted significant amounts of primordial molecular cloud material^91,92^. Their analysis reveals the occurrence of a ^26^Mg*-depleted component, interpreted to reflect the contribution from an r-process poor, cometary/interstellar component^92^.

From a dynamical point of view, it is unlikely that only one type of comet impacted the early Earth^79^, but the isotopic composition of noble gases within long-period comets akin to those now belonging to the Oort cloud (OCC) is unknown. Hence, whilst it cannot be stated with certainty that all comets (Kuiper belt objects and long period comets) have the same noble gas isotopic signature as comet 67P/C-G, it cannot be dismissed either, and the nucleosynthetically distinct nature of noble gases in comet 67P/C-G remains our best analogue to the signature of the bulk cometary reservoir. We note that most comets present comparable enrichments in D relative to H, as well as consistent enrichments in ^15^N relative to ^14^N^35^, suggesting that comets from the OCC and JFC both have sampled a common reservoir. Addressing the potential variability of noble gases within cometary material, and how this would affect our estimates of the cometary contribution to the terrestrial atmosphere, will require the analysis of new cometary materials belonging to different dynamical families, which would only be achievable though future spatial missions.

(b) We consider the elemental ratios of noble gases within sublimating cometary ice and measured within the coma of comet 67P/C-G to be representative of the bulk comet composition. Yet, phases other than ice (namely organic materials and silicates) may contribute the bulk composition of cometary noble gases. The highest content of ^132^Xe measured so far for the insoluble organic matter of a carbonaceous chondrite is 2.2.10^−11^ mol ^132^Xe/g (Paris meteorite^86^), which is 2–3 orders of magnitude lower than the concentration of ^132^Xe in cometary ice (~7.4.10^−9^ mol ^132^Xe/g)^31^. Heavy noble gas concentrations in silicate phases, as measured in meteorites, are orders of magnitude lower than those found in chondritic IOM. Hence, although some uncertainties remain regarding the dust to ice ratio of cometary materials, mass balance considerations clearly indicate that the noble gas composition of ice controls that of the bulk comet. Importantly, fractionation of cometary noble gases could potentially have occurred during their release from the cometary surface; discrepancies in the outgassing velocities between Ar, Kr and Xe were taken into account to derive the cometary ice elemental composition used in this study^31^.

Regarding chondrites, bulk elemental ratios used in the present study could have been modified from their initial values by several processes, including thermal metamorphism, aqueous alteration and cosmogenic isotope production. However, parent body processes resulting in larger depletions of Ar and Kr relative to Xe in chondrites only cause elemental ratios to vary within a maximum factor of ~3^33,52,86^. Interestingly, EC, which represent the most metamorphosed chondritic group, show the highest ^84^Kr/^132^Xe, indicating that the effect of thermal metamorphism has not been sufficient enough to overprint the higher ^84^Kr/^132^Xe of the trapped subsolar component^53^. Finally, the isotopes we use to compute elemental ratios (^36^Ar, ^84^Kr and ^132^Xe) are abundant and limitedly produced by spallation reactions (unlike e.g., ^38^Ar, ^83^Kr or ^124–128^Xe)^93^ so that the production of cosmogenic isotopes would not affect so much the considered elemental ratios. Unlike noble gas abundances and elemental ratios, noble gas to water ratios vary over several orders of magnitude difference between EC/OC and CC (Table 2). These variations are fundamentally controlled by the amount of water accreted within the respective parent bodies, reflecting a gradient in the heliocentric distance at which parent bodies of the different meteoritic groups accreted^5^. Although the Kr/Xe/water ratios measured in chondrites might not exactly represent the composition of materials supplied to Earth during planetary accretion, we consider their subsequent modification to have been sufficiently limited to permit their use in this study. In addition, note that no general consensus on the elimination procedure of adsorbed water, required to accurately estimate the indigenous water content of chondritic material, can be found in the literature. Our results, which rely on the several orders of magnitude difference in water content between EC/OC and CC, would however marginally be affected by potential water contamination issues.

At last, we note that some uncertainties remain regarding the degree of differentiation of the Earth’s accretionary building blocks^94^, so the use of bulk chondrite compositions in our mixing calculations may induce a bias in the resulting estimates of the mass of chondritic material being required to supply chondritic volatiles to the atmosphere. Likewise, heavy noble gases in the mantle, as determined from the analysis of popping rocks^46^ and CO_2_ well gases^47^, are chondritic in origin, so degassing of mantle-derived volatiles over geological periods of time could have significantly contributed the chondritic component in the atmosphere. It is not clear how much the late additions of chondritic material (e.g., as part of the late veneer) and the degassing of chondritic noble gases from the mantle, contributed to form the atmosphere, respectively. From mass balance considerations, a ~0.5 wt.% of the Earth late veneer contribution of chondritic material would not be sufficient in supplying chondritic noble gases to the atmosphere^6^, therefore requiring significant contribution from mantle degassing. However, the required mass of a late veneer being mainly composed of achondritic material that had experienced differentiation and volatile loss before accretion to Earth could be as high as a few wt.% of the Earth^94^. Although this is an interesting point that would merit further investigation, how the accretion of achondritic material to the Earth would affect the estimates presented in this contribution is ultimately unknown. Interestingly, integrating the present day accretion rate of cosmic dust^95^ with ~50 wt% H_2_O would bring <10^−4^ times less water than the present day mass of the oceans, indicating that the influx of cosmic dust likely played a marginal role in supplying terrestrial volatiles.

(c) Processes involved in enriching or depleting the various isotopes of Xe over geological timescales are not completely understood. The scenario we favour here (Xe escape to space) is so far the only viable mechanism accounting for both the isotopic composition and elemental depletion of Xe isotopes in the terrestrial atmosphere. It notably relies on the fact that, due to the enhancement of the EUV emission of the young Sun at 3.5 Ga, atmospheric Xe photoionization rate was increased by about 4000% (against ~1000% for lighter noble gases)^14^. However, the exact conditions allowing for such an isotopic evolution and loss of atmospheric Xe through escape may vary depending on the possible time evolution of, e.g., the terrestrial magnetic field, the stellar UV flux, and/or the H_2_ partial pressure in the atmosphere. One possibility is that Xe isotopic evolution was not as continuous and protracted as previously thought; for instance, it could have been restricted to polar windows opening within the geomagnetic field, outbursts of high solar activity, limited to transient episodes of abundant hydrogen, or a combination of all three^16^.

Likewise, we conclude in section 4 for the noble gas to water ratio of the ESR to have been established early during Earth’s history, with impact-driven loss of the early atmosphere constituting the main process driving noble gas to water fractionation of the ESR with respect to its precursor material composition. However, it should be noted that our knowledge of how planetary processes affected the budget of volatile elements within the ESR over geological periods of time suffers from several caveats that hamper definitive conclusions to be drawn from the considerations presented in this contribution. The extent of C^96^, N^97^ and noble gas^98^ recycling into the mantle is subject to debate and could have significantly altered the initial composition of the ESR. Better constraining the conditions and effects of the Moon-forming giant impact on the terrestrial budget of volatile elements, the extent of volatile transfers between the Earth’s interior and its surface during the subsequent magma ocean episode, the timing of the onset of subduction and volatile recycling into the solid Earth, as well as the composition and evolution of the primitive atmosphere, will ultimately allow the main type(s) of chondritic material that supplied the Earth surface with most of its volatile elements to be further elucidated.

## Methods

### Determining the composition of chondritic endmembers

In order to derive a representative concentration for a given element within a given type of chondrite (CC, OC and EC), we applied interquartile range calculations on data compiled from the literature (see Supplementary Information). Due to the fact that concentrations of volatiles within chondrites can vary across several orders of magnitude and are not necessarily normally distributed, the use of mean values and standard deviations to derive the average composition of each endmember may not be justified. In addition, extreme outliers might artificially influence the value of the mean, while keeping the median of the dataset unaffected. For this reason, we computed the median concentration of Ar, Kr, Xe, N, C and H_2_O within the three chondritic endmembers (CC, EC and OC) and determined statistical dispersion of the data by calculating the interquartile range (IQR). The IQR calculates the difference between the 25th (Q_1_) and the 75th (Q_3_) percentiles, i.e. IQR = Q_3_−Q_1_. The IQR provides a method for excluding extreme outliers and providing a range within which 50% of the data lie. In Table 2, we report values for the median, first (Q1) and third (Q3) quartiles of each data set (${{\rm{Median}}}_{Q3}^{Q1}$). The ranges of values we use for mixing calculations are taken as [Median − Q1; Median + Q3]. Interquartile ranges are robust measures of variability, in a similar manner that medians are robust measures of central tendency. Just like medians, interquartile ranges are excellent for avoiding large effects on potentially skewed distributions, which is not the case for means and standard deviations.

### Computing the extent of Xe loss

The first approach aims at determining the initial ^84^Kr/^132^Xe of the atmosphere by mixing cometary and chondritic end-members with known ^84^Kr/^132^Xe ratios. Using the ^84^Kr/^132^Xe of comets and chondrites (Table 1), we derive theoretical ^84^Kr/^132^Xe ratios for the initial atmosphere. These ratios are systematically lower than the present day atmospheric ^84^Kr/^132^Xe (Table 1), therefore requiring that part of the atmospheric ^132^Xe was lost. For each theoretical ^84^Kr/^132^Xe, we compute how many times the present-day budget of atmospheric ^132^Xe should be lost to fit the actual ^84^Kr/^132^Xe of the present-day atmosphere.

The second approach is based on the fact that ~22% of the atmospheric Kr and Xe are cometary in origin. We calculate the amount of Kr corresponding to 21 ± 5% of the present-day inventory of the ESR (Table 2). Using the ^84^Kr/^132^Xe ratio in cometary ice, we derive a theoretical budget of Xe that would have been brought to Earth by comets. This budget of Xe is compared to the actual amount of atmospheric Xe being cometary in origin in the ESR, i.e. 22 ± 5% (^12^; Table 2). We find that this theoretical budget of cometay Xe in the atmosphere is higher than the actual Xe budget of the present-day ESR. This is likely accounted for by the fact that part of atmospheric Xe has been lost to space, therefore yielding a present-day amount of Xe in the ESR being significantly lower than what had been initially accreted by early Earth. We therefore compute how many times the present-day budget of Xe in the ESR would be required to be added (i.e., corrected for its loss) to fit the cometary Xe budget of the primitive ESR.

### Computing the cometary contribution to terrestrial volatiles

Cometary contribution to the budget of Kr and Xe in the ESR is ~22%, with the remaining ~78% being derived from chondritic material^12^. Cometary contribution to the ESR budget of water (taken as an example here) can be computed if the noble gas to water elemental ratio is known for both cometary (referred to as “com.”) and chondritic (referred to as “ch.”) endmembers:

1 $$\frac{{{\rm{H}}}_{2}{{\rm{O}}}_{{\rm{com}}.}}{{{\rm{H}}}_{2}{{\rm{O}}}_{{\rm{ch}}.}}=\frac{{{\rm{H}}}_{2}{{\rm{O}}}_{{\rm{com}}.}}{{}^{132}{\rm{X}}{{\rm{e}}}_{{\rm{com}}.}}\,\ast \,\frac{{}^{132}{\rm{X}}{{\rm{e}}}_{{\rm{com}}.}}{{}^{132}{\rm{X}}{{\rm{e}}}_{{\rm{ch}}.}}\,\ast \,\frac{{}^{132}{\rm{X}}{{\rm{e}}}_{{\rm{ch}}.}}{{{\rm{H}}}_{2}{{\rm{O}}}_{{\rm{ch}}.}}$$

noted as: ${\rm{\beta }}={\rm{\alpha }}\,\ast \,{\rm{\gamma }}$with ${\rm{\alpha }}=\,\frac{{}^{132}{\rm{X}}{{\rm{e}}}_{{\rm{com}}.}}{{}^{132}{\rm{X}}{{\rm{e}}}_{{\rm{ch}}.}},{\rm{\beta }}=\frac{{{\rm{H}}}_{2}{{\rm{O}}}_{{\rm{com}}.}}{{{\rm{H}}}_{2}{{\rm{O}}}_{{\rm{ch}}.}}$ and $\,{\rm{\gamma }}=\,\frac{\frac{{{\rm{H}}}_{2}{{\rm{O}}}_{{\rm{com}}.}}{{}^{132}{\rm{X}}{{\rm{e}}}_{{\rm{com}}}.}}{\frac{{{\rm{H}}}_{2}{{\rm{O}}}_{{\rm{ch}}.}}{{}^{132}{\rm{X}}{{\rm{e}}}_{{\rm{ch}}.}}}$.

The cometary ^132^Xe/H_2_O is taken as that of comet 67P/C-G, therefore causing the γ parameter to be function of the chondritic ^132^Xe/H_2_O only. We note:

$${\rm{x}}=\frac{{}^{132}{\rm{X}}{{\rm{e}}}_{{\rm{com}}.}}{{}^{132}{\rm{X}}{{\rm{e}}}_{{\rm{ATM}}}},\,{\rm{y}}=\frac{{{\rm{H}}}_{2}{{\rm{O}}}_{{\rm{com}}.}}{{{\rm{H}}}_{2}{{\rm{O}}}_{{\rm{ATM}}}}\,{\rm{and}}\,{\rm{\alpha }}=\frac{{\rm{x}}}{1-{\rm{x}}},\,{\rm{\beta }}=\frac{{\rm{y}}}{1-{\rm{y}}}.$$

For each value of x, which is the fraction of cometary Xe in the atmosphere (taken from 0% to 100%), we calculate the corresponding value of α and derive β for given values of chondritic ^132^Xe/H_2_O (i.e., different values of ɣ). This allows the corresponding contribution of comets to the budget of terrestrial surface water (parameter y) to be calculated. The same calculations have been carried out using the noble gas to carbon, nitrogen and halogen elemental ratio for both cometary and chondritic end-members (Table 2).

### Computing the ^132^Xe/H_2_O of the chondritic component in the atmosphere

We establish here the equation giving the ^132^Xe/H_2_O of the initial atmosphere as a function of the cometary or chondritic ^132^Xe/H_2_O. We have:

2 $${\left(\frac{{}^{132}{\rm{X}}{\rm{e}}}{{{\rm{H}}}_{2}{\rm{O}}}\right)}_{{\rm{ESR}},{\rm{model}}}=\frac{{}^{132}{\rm{X}}{{\rm{e}}}_{{\rm{ESR}}}}{{{\rm{H}}}_{2}{{\rm{O}}}_{{\rm{ESR}}}}=\frac{{}^{132}{\rm{X}}{{\rm{e}}}_{{\rm{com}}.}+{}^{132}{\rm{X}}{{\rm{e}}}_{{\rm{ch}}.}}{{{\rm{H}}}_{2}{{\rm{O}}}_{{\rm{com}}.}+{{\rm{H}}}_{2}{{\rm{O}}}_{{\rm{ch}}.}}$$

which equals to:

3 $${\left(\frac{{}^{132}{\rm{X}}{\rm{e}}}{{{\rm{H}}}_{2}{\rm{O}}}\right)}_{{\rm{ESR}},{\rm{model}}}=\frac{{}^{132}{\rm{X}}{{\rm{e}}}_{{\rm{com}}.}}{{{\rm{H}}}_{2}{{\rm{O}}}_{{\rm{com}}}.}\,\ast \,\frac{\,\left(1\,+\frac{{}^{132}{\rm{X}}{{\rm{e}}}_{{\rm{ch}}.}}{{}^{132}{\rm{X}}{{\rm{e}}}_{{\rm{com}}.}}\right)}{\,\left(1\,+\frac{{{\rm{H}}}_{2}{{\rm{O}}}_{{\rm{ch}}.}}{{{\rm{H}}}_{2}{{\rm{O}}}_{{\rm{com}}.}}\right)}=\frac{{}^{132}{\rm{X}}{{\rm{e}}}_{{\rm{com}}.}}{{{\rm{H}}}_{2}{{\rm{O}}}_{{\rm{com}}.}}\,\ast \,\frac{\,(1\,+1/{\rm{\alpha }})}{\,(1\,+1/{\rm{\beta }})}$$

4 $${\left(\frac{{}^{132}{\rm{X}}{\rm{e}}}{{{\rm{H}}}_{2}{\rm{O}}}\right)}_{{\rm{ESR}},{\rm{model}}}=\frac{{}^{132}{\rm{X}}{{\rm{e}}}_{{\rm{ch}}.}}{{{\rm{H}}}_{2}{{\rm{O}}}_{{\rm{ch}}.}}\,\ast \,\frac{\,\left(\frac{{}^{132}{\rm{X}}{{\rm{e}}}_{{\rm{com}}.}}{{}^{132}{\rm{X}}{{\rm{e}}}_{{\rm{ch}}.}}+1\right)}{\,\left(\frac{{{\rm{H}}}_{2}{{\rm{O}}}_{{\rm{com}}.}}{{{\rm{H}}}_{2}{{\rm{O}}}_{{\rm{ch}}.}}+1\right)}=\frac{{}^{132}{\rm{X}}{{\rm{e}}}_{{\rm{ch}}.}}{{{\rm{H}}}_{2}{{\rm{O}}}_{{\rm{ch}}.}}\,\ast \,\frac{({\rm{\alpha }}+1)}{({\rm{\beta }}+1)}$$

By considering a constant mass of water within the ESR since the last major episode of impact erosion of the atmosphere, which is presumably the Moon forming event, the ESR can be corrected for its loss of Xe through atmospheric escape in order to derive theoretical ^132^Xe/H_2_O for the primitive atmosphere. In Eq. (4), we note that the initial ^132^Xe/H_2_O of the ESR only depends on the ^132^Xe/H_2_O of the chondritic end-member. A theoretical value for the ^132^Xe/H_2_O of the chondritic component in the atmosphere can therefore be calculated for any given extent of Xe loss, with:

5 $$AT{M}_{Xe}lost=\frac{{\left(\frac{{}^{132}{\rm{X}}{\rm{e}}}{{{\rm{H}}}_{2}{\rm{O}}}\right)}_{{\rm{ESR}},{\rm{model}}}}{{\left(\frac{{}^{132}{\rm{X}}{\rm{e}}}{{{\rm{H}}}_{2}{\rm{O}}}\right)}_{{\rm{ESR}},{\rm{observed}}}}=\frac{\frac{{}^{132}{\rm{X}}{{\rm{e}}}_{{\rm{ch}}.}}{{{\rm{H}}}_{2}{{\rm{O}}}_{{\rm{ch}}.}}\,\ast \,\frac{({\rm{\alpha }}+1)}{({\rm{\beta }}+1)}}{{\left(\frac{{}^{132}{\rm{X}}{\rm{e}}}{{{\rm{H}}}_{2}{\rm{O}}}\right)}_{{\rm{ESR}},{\rm{observed}}}}$$

### Computing the maximum mass of comets striking the Earth after the Moon-forming impact

Due to the fact that part of the volatile elements accreted during the later stages of accretion may have been lost through impact erosion of the Earth surface, the total mass of comets that struck the Earth over its accretionary history cannot be determined. However, because the loss of Kr from the Earth surface reservoir is thought to have been limited since the Moon-forming giant impact, the maximum mass of cometary materials that were accreted on Earth after this episode can be calculated from the amount of cometary Kr now residing in the ESR. We use the 22% cometary ^84^Kr in the ESR to derive the corresponding amount of water ice that would have been brought by comets based on the ^84^Kr/H_2_O ratio of comet 67 P/C-G (Table 2). We find that the maximum amount of water that would have been brought by comets along with cometary Kr after the Moon-forming impact is ~1.08 × 10^20^ mol H_2_O (~1.95 × 10^21^ g H_2_O). According to Pätzold *et al*. (2016), the most likely composition mix of comet 67 P/C-G has approximately four times more dust than ice by mass, with a ratio of 1:1 for organic carbonaceous particles and silicates. The cometary ice composition is at least ~80 wt.% H_2_O, with the remaining 20 wt.% being mainly composed with CO, CO_2_, CH_3_OH, CH_4_, H_2_S and N-compounds including CN, HCN, NH_3_ and N^18^. In a first approximation, we assume that cometary ice is 100 wt.% H_2_O and use the total mass of cometary water being brought to Earth (~1.95 × 10^21^ g H_2_O) to derive the total mass of comets accreted by Earth (~9.76 × 10^21^ g). This equals ~0.03 wt.% of the late veneer, taken as 0.5 wt.% of the Earth, and would correspond to a single sphere with a diameter of ~340 km with a density of 0.5 g/cm^3^ ^41^. Along with water, we estimate that comets could have brought up to 4 × 10^21^g of organic materials.

## Supplementary information

Supplementary information 1.

Supplementary information 2.
