# Supplementary material for: The origin and fate of volatile elements on Earth revisited in light of noble gas data obtained from comet 67P/Churyumov-Gerasimenko
Source: Sci Rep. 2020 Apr 2;10:5796. doi: 10.1038/s41598-020-62650-3 (PMC7118078; doi:10.1038/s41598-020-62650-3)
Supplement: Supplementary file 2 — Supplementary information 2. [file 41598_2020_62650_MOESM2_ESM.pdf]

The origin and fate of volatile elements on Earth revisited in light of noble gas data obtained from comet 67P/Churyumov-Gerasimenko

David V. Bekaert[1]*; Michael W. Broadley[1]*; Bernard Marty[1]

Supplementary Information

S1° Determining the composition of chondritic endmembers (excel file)

S2° Literature data used to construct Table 2.

S3° Computing the contribution of cometary krypton in the atmosphere.

Fig. S1° Kr three isotope plots refute a significant contribution from solar Kr to the atmosphere.

Fig. S2° Kr three isotope plots: Kr-N and Kr-G mixtures to reproduce the cometary signature.

Fig. S3° Percentage of cometary nitrogen accreted to Earth and the resulting effect on the composition of the Earth's surface.

Fig. S4° Efficient aerobracking of low-density cometary material upon entry in the atmosphere.

S1° Determining the composition of chondritic endmembers

(see excel file attached, "compilation-SR-Bekaert.xlsx")

S2° Literature data used to construct Table 2.

Calculations for volatile element concentrations in CC, OC and EC are reported in the excel file provided as a Supplementary material ("compilation-SR-Bekaert.xls"). References used to calculate the IQR for each volatiles species is reported here below.

	CC	OC	EC
^{36}Ar		Schelhaas et al. (1990)[7]<br>Heymann and Mazor (1968)[8]<br>Hashizume and Sugiura (1995)[9]	
^{84}Kr	Mazor et al. (1970)[1]<br>Nakamura (2006)[2]<br>Bekaert et al. (2019)[3]<br>Haack et al. (2012)[4]<br>Okazaki and Nagao (2017)[5]<br>Srinivasan et al. (1978)[6]	Schelhaas et al. (1990)[7]<br>Heymann and Mazor (1968)[8]<br>Alaerts et al. (1979)[10]	Patzer and Schultz (2002)[11]<br>Okazaki et al. (2010)[12]<br>Crabb and Anders (1982)[13]
^{132}Xe		Schelhaas et al. (1990)[7]<br>Heymann and Mazor (1968)[8]<br>Alaerts et al. (1979)[10]	
H_2O	Jarosewich (1990)[14]<br>Piani et al. (2018)[15]	Jarosewich (1990)[14]	Javoy (1995)[16]<br>Jarosewich (1990)[14]
^{12}C	Alexander et al. (1990)[17]<br>Kerridge (1985)[18]<br>Jarosewich (1990)[14]<br>Piani et al. (2018)[15]	Grady et al. (1986)[18]<br>Jarosewich (1990)[14]	Jarosewich (1990)[14]
^{14}N	Kerridge (1985)[19]<br>Kung and Clayton (1978)[20]	Kung and Clayton (1978)[19]<br>Hashizume and Sugiura (1995)[9]	Grady et al. (1986)[18]<br>Kung and Clayton (1978)[19]

Given that the ^{84}Kr/^{132}Xe of enstatite chondrites defines a unique component (Q + subsolar), we take the standard deviation to compute the average value and range of ^{84}Kr/^{132}Xe in this mixture. The mean ^{84}Kr/^{132}Xe of enstatite chondrites (=1.84 ± 1.14; Table 1) is calculated by taking the mean and standard deviation of all ^{84}Kr/^{132}Xe (n=73) reported by [11,12,13]. The choice of IQR to define the range of ^{84}Kr/^{132}Xe in enstatites is not necessarily justified given that all data points lie on a mixing line between these two components. Nevertheless the choice of IQR to calculate the ^{84}Kr/^{132}Xe of enstatite components would not critically change our conclusions (1.84±1.14 for SD vs. $1.6^{0.7}_{1.0}$ for IQR).

Halogen (^{35}Cl, ^{79}Br, ^{127}I) concentrations in chondritic endmembers and in terrestrial reservoirs (ESR and BM), as well as the mass of the respective terrestrial reservoirs, are from [21]. ^{19}F concentrations in chondrites are from [22]. Halogens concentrations in cometary ice are from [23] for ^{35}Cl, ^{19}F and ^{79}Br, and from [24] for ^{127}I.

Noble gas, water, nitrogen and carbon concentration in the ESR and bulk mantle are from compilation by [25]. δ^{15}N(‰) values for chondritic endmembers and bulk comets are from [26] and [27], respectively. Noble gas concentrations in cometary ice are from [28-29]. Water content and dust to ice ratio of cometary material (used to compute the concentrations of volatile elements in cometary matter) is from [30]. δD(‰) values for each chondritic endmember are from compilation by [31], with the range of cometary δD(‰) values given by [27].

S3° Computing the contribution of cometary krypton in the atmosphere

Mixtures were calculated for each Kr isotopes by taking 10^5 isotopic compositions ($^{i}Kr/^{84}Kr$) from the normal error distribution of comet 67P/C-G[29], chondritic (Q)[32], and atmospheric Kr[33] (Table S1).

Component	$^{80}Kr/^{84}Kr$	±	$^{82}Kr/^{84}Kr$	±	$^{83}Kr/^{84}Kr$	±	$^{84}Kr/^{84}Kr$	$^{86}Kr/^{84}Kr$	±
**Atmosphere**	3.96	0.02	20.217	0.021	20.136	0.021	=100	30.524	0.025
**Q-Kr**	3.937	0.07	20.18	0.02	20.18	0.02	=100	30.95	0.05
**67P/C-G**	3.87	0.38	20.30	0.44	18.73	0.15	=100	28.87	0.38

Table S3. Compilation of Kr isotopic ratios for each reservoir used to calculate cometary Kr contributions to the atmosphere. The Kr isotopic data for comet 67P/C-G is the average of three measurements[29], with the associated standard deviation. All ratios are normalised to ^{84}Kr and uncertainties are given to 1σ.

The contribution of cometary Kr to the atmosphere is given by:

$$\%\left(\frac{^{i}Kr}{^{84}Kr}\right)_{com-Kr} = \frac{\left(\frac{^{i}Kr}{^{84}Kr}\right)_{chond-Kr} - \left(\frac{^{i}Kr}{^{84}Kr}\right)_{atm-Kr}}{\left(\frac{^{i}Kr}{^{84}Kr}\right)_{chond-Kr} - \left(\frac{^{i}Xe}{^{84}Kr}\right)_{com-Kr}}$$

where $(^{i}Kr/^{84}Kr)_{chond}$, $(^{i}Kr/^{84}Kr)_{atm}$ and $(^{i}Kr/^{84}Kr)_{com}$ are the $^{i}Kr/^{84}Kr$ ratio (i = $^{80\text{-}84}Kr$) of chondritic-Kr, atmospheric-Kr and cometary-Kr respectively. The error weighted mean cometary contribution to the atmosphere is the calculated by:

$$\%(com-Kr) = \frac{\Sigma i\left(\frac{\%\left(\frac{^{i}Kr}{^{84}Kr}\right)_{com-Kr}}{\sigma^2{}_{\%\left(\frac{^{i}Kr}{^{84}Kr}\right)_{com-Kr}}}\right)}{\Sigma i\left(\frac{1}{\sigma^2{}_{\%\left(\frac{^{i}Kr}{^{84}Kr}\right)_{com-Kr}}}\right)}$$

with σ^2 given by:

$$\sigma^2{}_{\%(com-Kr)} = \frac{1}{\Sigma i\left(\frac{1}{\sigma^2{}_{\%\left(\frac{^{i}Kr}{^{84}Kr}\right)_{com-Kr}}}\right)}$$

where $\%(^{i}Kr/^{84}Kr)_{com\text{-}Kr}$ and $\sigma^2{}_{\%}(^{i}Kr/^{84}Kr)_{com\text{-}Kr}$ are the mean cometary contribution to the modern atmosphere and the associated error for each Kr isotopic ratio, respectively. The calculated cometary contribution to a Q atmosphere is 21 ± 5%.

Fig. S1° Kr three isotope plots refute a significant contribution from solar Kr to the atmosphere.

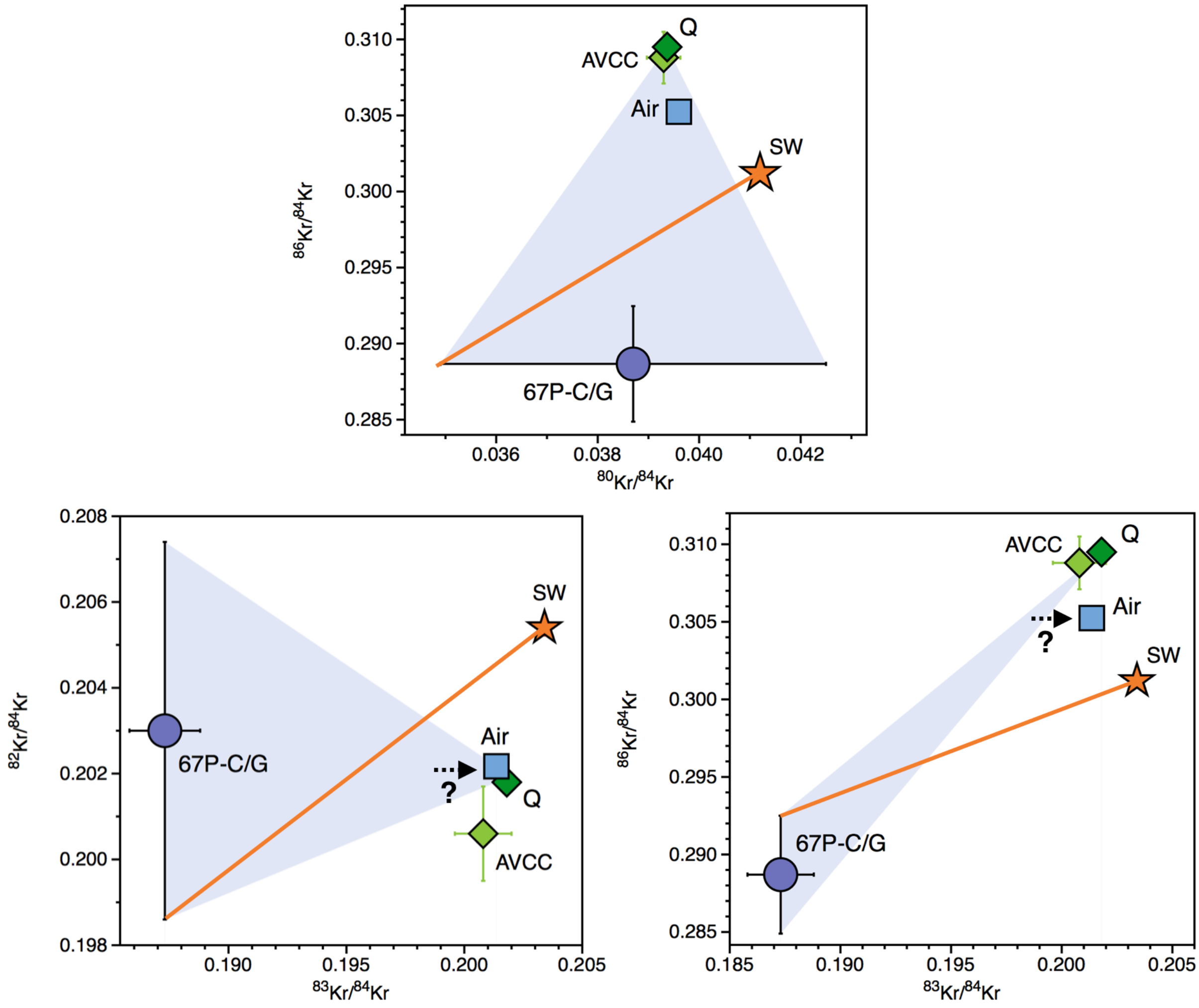

Fig. S2° Kr three isotope plots: Kr-N and Kr-G mixtures to reproduce the cometary signature. Percentages correspond to the fractions of G-Kr.

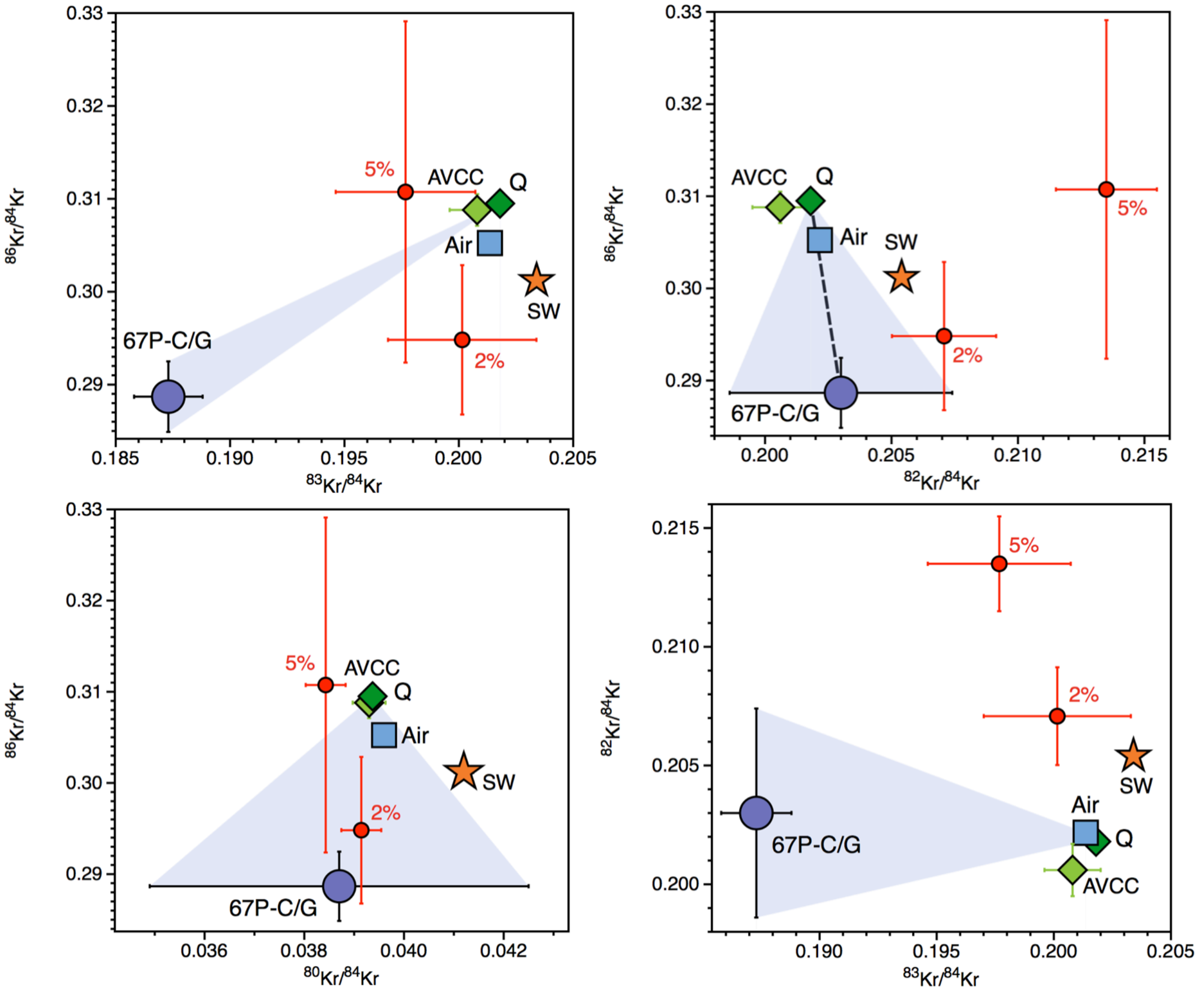

Fig. S3. Percentage of cometary nitrogen accreted to Earth and the resulting effect on the composition of the Earth's surface

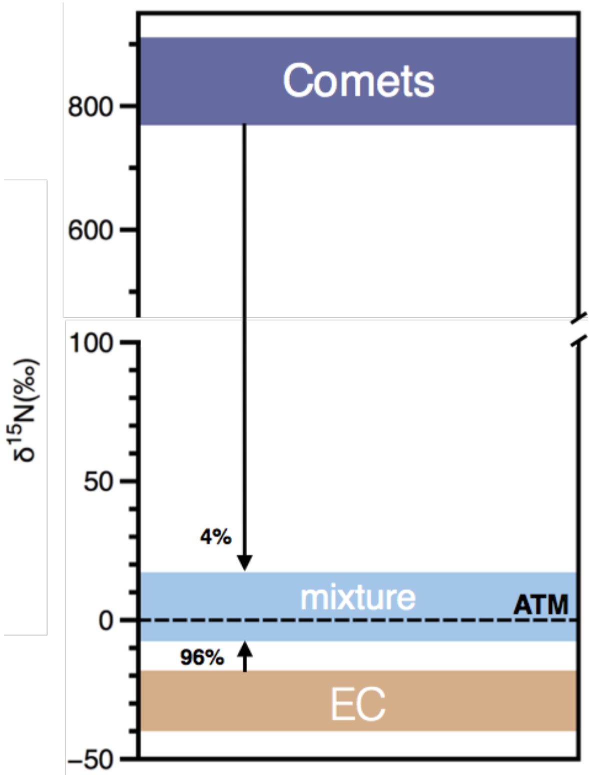

The mass of comets inferred to have been supplied to Earth would only have brought ~5% of the N in the atmosphere. Yet, the large ^{15}N enrichment of comets[27] may still have significantly raised the EC-like $\delta^{15}N$ found in the mantle[34] towards modern day atmospheric values.

Fig. S4° Efficient aerobraking of low-density cometary material upon entry in the atmosphere.

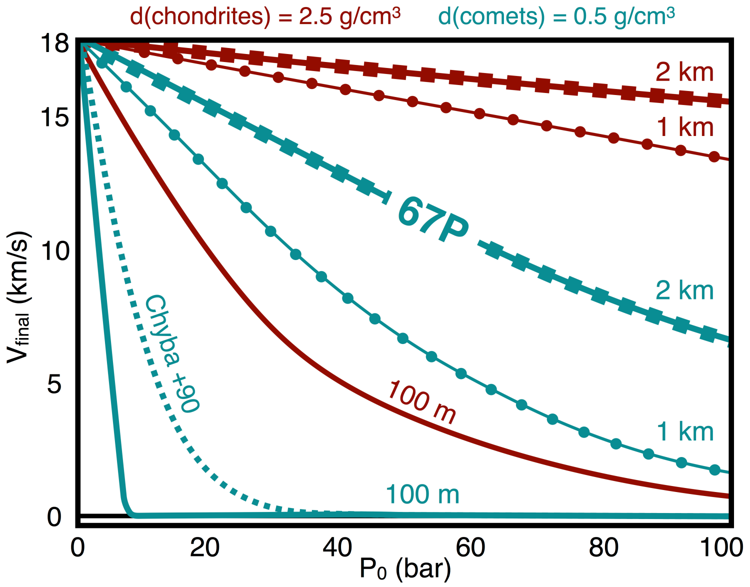

This figure corresponds to Fig. 1 from Chyba et al. (1990), modified using new cometary data from comet 67P/C-G[30]. Impact velocities are displayed as a function of surface atmospheric pressure for cometary (in green) and chondritic (in brown) impactors Incidence angle is taken as 0°, with radii defined as 100, 1000 and 2000m. The density of comet 67P/C-G has been determined to be low[30] ($0.5 g/cm^3$ instead of e.g. $1 g/cm^3$, as used by Chyba et al. (1990)). This allows cometary impactors with radii of 100 m to be efficiently aerobraked (see the "Chyba +90" dashed green curve relative to the solid green curve adapted to comet 67P/C-G) and effectively reach the surface of the primitive Earth with low impact velocities. Note however that this requires high atmospheric pressures (≥ 5 bar), which might not have been the case in the Archean[36].

Supplementary information references

1. Mazor, E., Heymann, D., & Anders, E. Noble gases in carbonaceous chondrites. *Geochimica et Cosmochimica Acta*, *34*(7), 781-824 (1970).
2. Nakamura, T. (2006). Yamato 793321 CM chondrite: Dehydrated regolith material of a hydrous asteroid. *Earth and Planetary Science Letters*, *242*(1-2), 26-38.
3. Bekaert, D. V., Marrocchi, Y., Meshik, A., Remusat, L., & Marty, B. (2019). Primordial heavy n
4. Haack, H., Grau, T., Bischoff, A., Horstmann, M., Wasson, J., Sørensen, A., ... & Greenwood, R. C. (2012). Maribo—A new CM fall from Denmark. *Meteoritics & Planetary Science*, *47*(1), 30-50.
5. Okazaki, R., & Nagao, K. (2017). Primordial and cosmogenic noble gases in the Sutter's Mill CM chondrite. *Meteoritics & Planetary Science*, *52*(4), 669-689.
6. Srinivasan, B., Lewis, R. S., & Anders, E. (1978). Noble gases in the Allende and Abee meteorites and a gas-rich mineral fraction: investigation by stepwise heating. Geochimica et Cosmochimica Acta, 42(2), 183-198.
7. Schelhaas, N., Ott, U., & Begemann, F. Trapped noble gases in unequilibrated ordinary chondrites. *Geochimica et Cosmochimica Acta*, *54*(10), 2869-2882 (1990).
8. Heymann, D., & Mazor, E. Noble gases in unequilibrated ordinary chondrites. *Geochimica et Cosmochimica Acta*, *32*(1), 1-19. (1968).
9. Hashizume, K., & Sugiura, N. Nitrogen isotopes in bulk ordinary chondrites. *Geochimica et cosmochimica acta*, *59*(19), 4057-4069 (1995).
10. Alaerts, L., Lewis, R. S., & Anders, E. Isotopic anomalies of noble gases in meteorites and their origins—III. LL-chondrites. *Geochimica et Cosmochimica Acta*, *43*(9), 1399-1415 (1979).
11. Patzer, A., & Schultz, L. Noble gases in enstatite chondrites II: The trapped component. *Meteoritics & Planetary Science*, *37*(4), 601-612 (2002).
12. Okazaki, R., Takaoka, N., Nagao, K., & Nakamura, T. Noble gases in enstatite chondrites released by stepped crushing and heating. *Meteoritics & Planetary Science*, *45*(3), 339-360 (2010).
13. Crabb, J., & Anders, E. (1982). On the siting of noble gases in E-chondrites. *Geochimica et Cosmochimica Acta*, *46*(11), 2351-2361.
14. Jarosewich, E. Chemical analyses of meteorites: A compilation of stony and iron meteorite analyses. *Meteoritics*, *25*(4), 323-337 (1990).
15. Piani, L., Yurimoto, H., & Remusat, L. A dual origin for water in carbonaceous asteroids revealed by CM chondrites. *Nature Astronomy*, 1 (2018).
16. Javoy, M. The integral enstatite chondrite model of the Earth. *Geophysical Research Letters*, *22*(16), 2219-2222 (1995).
17. Alexander C. M. O'D., Arden J. W., Ash R. D., and Pillinger C. T. Presolar components in the ordinary chondrites. Earth. Planet. Sci. Lett. 99, 220-229 (1990).
18. Grady, M. M., Wright, I. P., Carr, L. P., & Pillinger, C. T. Compositional differences in enstatite chondrites based on carbon and nitrogen stable isotope measurements. *Geochimica et Cosmochimica Acta*, *50*(12), 2799-2813 (1986).
19. Kerridge, J. F. Carbon, hydrogen and nitrogen in carbonaceous chondrites: Abundances and isotopic compositions in bulk samples. *Geochimica et Cosmochimica Acta*, *49*(8), 1707-1714 (1985).
20. Kung, C. C., & Clayton, R. N. Nitrogen abundances and isotopic compositions in stony meteorites. *Earth and Planetary Science Letters*, *38*(2), 421-435 (1978).

21. Clay, P. L., Burgess, R., Busemann, H., Ruzié-Hamilton, L., Joachim, B., Day, J. M., & Ballentine, C. J. Halogens in chondritic meteorites and terrestrial accretion. *Nature*, *551*(7682), 614 (2017).
22. Brearley, A. J., & Jones, R. H. Halogens in chondritic meteorites. In *The Role of Halogens in Terrestrial and Extraterrestrial Geochemical Processes* (pp. 871-958). Springer, Cham. (2018).
23. Dhooghe, F., De Keyser, J., Altwegg, K., Briois, C., Balsiger, H., Berthelier, J. J., ... & Fiethe, B. Halogens as tracers of protosolar nebula material in comet 67P/Churyumov–Gerasimenko. *Monthly Notices of the Royal Astronomical Society*, *472*(2), 1336-1345 (2017).
24. Marty, B. et al. Xenon isotopes in 67P/Churyumov-Gerasimenko show that comets contributed to Earth's atmosphere. *Science*, *356*(6342), 1069-1072 (2017).
25. Marty, B. The origins and concentrations of water, carbon, nitrogen and noble gases on Earth. *Earth and Planetary Science Letters*, *313*, 56-66 (2012).
26. Füri, E., & Marty, B. Nitrogen isotope variations in the Solar System. *Nature Geoscience*, *8*(7), 515 (2015).
27. Jehin, E., Manfroid, J., Hutsemekers, D., Arpigny, C., & Zucconi, J. M. (2009). Isotopic ratios in comets: status and perspectives. *Earth, Moon, and Planets*, *105*(2-4), 167-180.
28. Marty, B., Avice, G., Sano, Y., Altwegg, K., Balsiger, H., Hässig, M., ... & Rubin, M. Origins of volatile elements (H, C, N, noble gases) on Earth and Mars in light of recent results from the ROSETTA cometary mission. *Earth and Planetary Science Letters*, *441*, 91-102 (2016).
29. Rubin, M., Altwegg, K., Balsiger, H., Bar-Nun, A., Berthelier, J. J., Briois, C., ... & Fuselier, S. A. Krypton isotopes and noble gas abundances in the coma of comet 67P/Churyumov-Gerasimenko. *Science advances*, *4*(7), eaar6297 (2018).
30. Pätzold, M., Andert, T., Hahn, M., Asmar, S. W., Barriot, J. P., Bird, M. K., ... & Weissman, P. R. A homogeneous nucleus for comet 67P/Churyumov–Gerasimenko from its gravity field. *Nature*, *530*(7588), 63 (2016).
31. Barnes, J. J., Kring, D. A., Tartèse, R., Franchi, I. A., Anand, M., & Russell, S. S. An asteroidal origin for water in the Moon. *Nature communications*, *7*, 11684 (2016).
32. Busemann, H., Baur, H., & Wieler, R. Primordial noble gases in “phase Q” in carbonaceous and ordinary chondrites studied by closed-system stepped etching. *Meteoritics & Planetary Science*, *35*(5), 949-973 (2000).
33. Ozima M. and Podosek F. A. Noble gas geochemistry. Cambridge, UK: Cambridge University Press (2002).
34. Cartigny, P., Boyd, S., Harris, J., & Javoy, M. Nitrogen isotopes in peridotitic diamonds from Fuxian, China: the mantle signature. *Terra Nova*, *9*(4), 175-179 (1997).
35. Chyba, C. F., Thomas, P. J., Brookshaw, L., & Sagan, C. Cometary delivery of organic molecules to the early Earth. *Science*, *249*(4967), 366-373 (1990).
36. Marty, B., Zimmermann, L., Pujol, M., Burgess, R., & Philippot, P. Nitrogen isotopic composition and density of the Archean atmosphere. *Science*, *342*(6154), 101-104 (2013).
